# SARS-CoV-2 Disrupts Splicing, Translation, and Protein Trafficking to Suppress Host Defenses

**DOI:** 10.1016/j.cell.2020.10.004

**Published:** 2020-11-25

**Authors:** Abhik K. Banerjee, Mario R. Blanco, Emily A. Bruce, Drew D. Honson, Linlin M. Chen, Amy Chow, Prashant Bhat, Noah Ollikainen, Sofia A. Quinodoz, Colin Loney, Jasmine Thai, Zachary D. Miller, Aaron E. Lin, Madaline M. Schmidt, Douglas G. Stewart, Daniel Goldfarb, Giuditta De Lorenzo, Suzannah J. Rihn, Rebecca M. Voorhees, Jason W. Botten, Devdoot Majumdar, Mitchell Guttman

**Affiliations:** 1 Division of Biology and Biological Engineering, California Institute of Technology, Pasadena, CA 91125, USA; 2 Keck School of Medicine, University of Southern California, Los Angeles, CA 90089, USA; 3 Departments of Medicine, Division of Immunobiology and Microbiology, and Molecular Genetics, Larner College of Medicine, University of Vermont, Burlington, VT 05405, USA; 4 David Geffen School of Medicine, University of California, Los Angeles, Los Angeles, CA 90095, USA; 5 MRC-University of Glasgow Centre for Virus Research (CVR), Glasgow G61 1QH, UK; 6 Department of Surgery and University of Vermont Cancer Center, University of Vermont College of Medicine, 89 Beaumont Avenue, Burlington, VT 05405, USA; 7 Broad Institute of MIT and Harvard, Cambridge, MA 02142, USA

**Keywords:** SARS-CoV-2, RNA-protein interactions, NSP1, NSP8, NSP9, NSP16, interferon, mRNA splicing, translation, protein trafficking

## Abstract

Severe acute respiratory syndrome coronavirus 2 (SARS-CoV-2) is a recently identified coronavirus that causes the respiratory disease known as coronavirus disease 2019 (COVID-19). Despite the urgent need, we still do not fully understand the molecular basis of SARS-CoV-2 pathogenesis. Here, we comprehensively define the interactions between SARS-CoV-2 proteins and human RNAs. NSP16 binds to the mRNA recognition domains of the U1 and U2 splicing RNAs and acts to suppress global mRNA splicing upon SARS-CoV-2 infection. NSP1 binds to 18S ribosomal RNA in the mRNA entry channel of the ribosome and leads to global inhibition of mRNA translation upon infection. Finally, NSP8 and NSP9 bind to the 7SL RNA in the signal recognition particle and interfere with protein trafficking to the cell membrane upon infection. Disruption of each of these essential cellular functions acts to suppress the interferon response to viral infection. Our results uncover a multipronged strategy utilized by SARS-CoV-2 to antagonize essential cellular processes to suppress host defenses.

## Highlights

• NSP16 binds mRNA recognition domains of U1/U2 snRNAs and disrupts mRNA splicing
• NSP1 binds in the mRNA entry channel of the ribosome to disrupt protein translation
• NSP8 and NSP9 bind the signal recognition particle and disrupt protein trafficking
• These disruptions of protein production suppress the interferon response to infection

## Graphical Abstract

**Figure:**
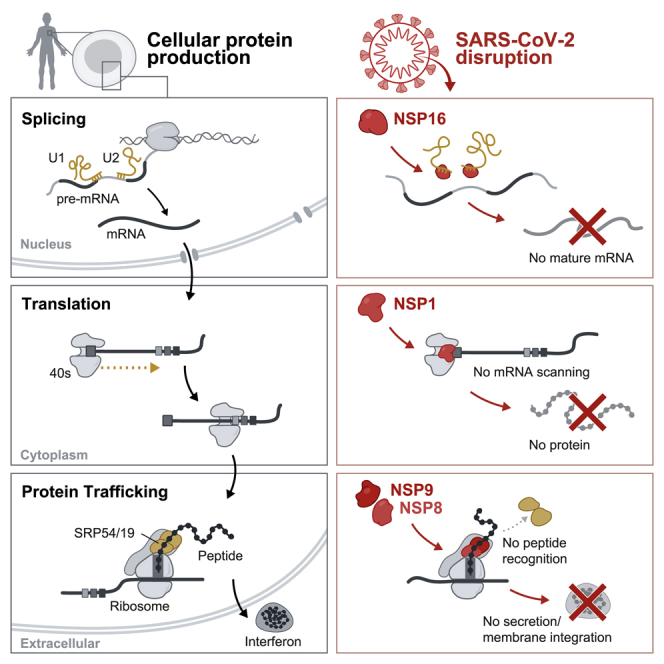

## Teaser

SARS-CoV-2 proteins directly engage host RNAs to dysregulate essential steps of protein production and suppress the interferon response.

## Introduction

Coronaviruses are a family of viruses with notably large single-stranded RNA genomes and broad species tropism among mammals (Graham and Baric, 2010). Recently, a coronavirus, severe acute respiratory syndrome coronavirus 2 (SARS-CoV-2), was discovered to cause the severe respiratory disease known as coronavirus disease 2019 (COVID-19). It is highly transmissible in human populations, and its spread has resulted in a global pandemic with more than a million deaths to date (Andersen et al., 2020; Zou et al., 2020). We do not fully understand the molecular basis of infection and pathogenesis of this virus in human cells. Accordingly, there is an urgent need to understand these mechanisms to guide the development of therapeutic agents.

SARS-CoV-2 encodes 27 proteins with diverse functional roles in virus replication and packaging (Bar-On et al., 2020; Wang et al., 2020). These include 4 structural proteins: the nucleocapsid (N; which binds the viral RNA) and the envelope (E), membrane (M), and spike (S) proteins, which are integral membrane proteins. In addition, there are 16 non-structural proteins (NSP1–NSP16) that encode the RNA-directed RNA polymerase, helicase, and other components required for virus replication (da Silva et al., 2020). Finally, there are 7 accessory proteins (ORF3a–ORF8) whose function in virus replication or packaging remains largely uncharacterized (Chen and Zhong, 2020; Finkel et al., 2020).

As obligate intracellular parasites, viruses require host cell components to translate and transport their proteins and to assemble and secrete viral particles (Maier et al., 2016). The mammalian innate immune system acts to rapidly detect and block viral infection at all stages of the virus life cycle (Chow et al., 2018; Jensen and Thomsen, 2012; Wilkins and Gale, 2010). The primary form of intracellular virus surveillance engages the interferon pathway, which amplifies signals resulting from detection of intracellular viral components to induce a systemic type I interferon response upon infection (Stetson and Medzhitov, 2006). Specifically, cells contain various RNA sensors (such as RIG-I and MDA5) that detect the presence of viral RNAs and promote nuclear translocation of the transcription factor IRF3, leading to transcription, translation, and secretion of interferon (e.g., interferon [IFN]-α and IFN-β). Binding of IFN to cognate cell-surface receptors leads to transcription and translation of hundreds of antiviral genes.

In order to successfully replicate, viruses employ a range of strategies to counter host antiviral responses (Beachboard and Horner, 2016). In addition to their essential roles in the viral life cycle, many viral proteins also antagonize core cellular functions in human cells to evade host immune responses. For example, human cytomegalovirus (HCMV) encodes proteins that inhibit major histocompatibility complex (MHC) class 1 display on the cell surface by retaining MHC proteins in the endoplasmic reticulum (Miller et al., 1998), polioviruses encode proteins that degrade translation initiation factors (eIF4G) to prevent translation of 5′-capped host mRNAs (Kempf and Barton, 2008; Lloyd, 2006), and influenza A encodes a protein that modulates mRNA splicing to degrade the mRNA that encodes RIG-I (Kochs et al., 2007; Zhang et al., 2018).

Suppression of the IFN response has recently emerged as a major clinical determinant of COVID-19 severity (Zhang et al., 2020), with almost complete loss of secreted IFN characterizing the most severe cases (Hadjadj et al., 2020). The extent to which SARS-CoV-2 suppresses the IFN response is a key characteristic that distinguishes COVID-19 from SARS and Middle East respiratory syndrome (MERS) (Lokugamage et al., 2020). Several strategies have been proposed for how the related SARS- and MERS-causing viruses may hijack host cell machinery and evade immune detection, including repression of host mRNA transcription in the nucleus (Canton et al., 2018), degradation of host mRNA in the nucleus and cytoplasm (Kamitani et al., 2009; Lokugamage et al., 2015), and inhibition of host translation (Nakagawa et al., 2018). Nonetheless, the extent to which SARS-CoV-2 uses these or other strategies and how they may be executed at a molecular level remains unclear.

Understanding the interactions between viral proteins and components of human cells is essential for elucidating their pathogenic mechanisms and for development of effective therapeutic agents. Because SARS-CoV-2 is an RNA virus, and many of its encoded proteins are known to bind RNA (Sola et al., 2011), we reasoned that these viral proteins may interact with specific human mRNAs (critical intermediates in protein production) or non-coding RNAs (critical structural components of diverse cellular machines) to promote virus propagation.

Here we comprehensively define the interactions between each SARS-CoV-2 protein and human RNA. We show that 10 viral proteins form highly specific interactions with mRNAs or noncoding RNAs (ncRNAs), including those involved in progressive steps of host cell protein production. We show that NSP16 binds to the mRNA recognition domains of the U1 and U2 RNA components of the spliceosome and acts to suppress global mRNA splicing in SARS-CoV-2-infected human cells. We find that NSP1 binds to a precise region on the 18S ribosomal RNA that resides in the mRNA entry channel of the initiating 40S ribosome. This interaction leads to global inhibition of mRNA translation upon SARS-CoV-2 infection of human cells. Finally, we find that NSP8 and NSP9 bind to discrete regions on the 7SL RNA component of the signal recognition particle (SRP) and interfere with protein trafficking to the cell membrane upon infection. We show that disruption of each of these essential cellular functions acts to suppress the type I IFN response to viral infection. Our results uncover a multipronged strategy utilized by SARS-CoV-2 to antagonize essential cellular processes and robustly suppress host immune defenses.

## Results

### Comprehensive Mapping of SARS-CoV-2 Protein Binding to Human RNAs

**Figure S1:**
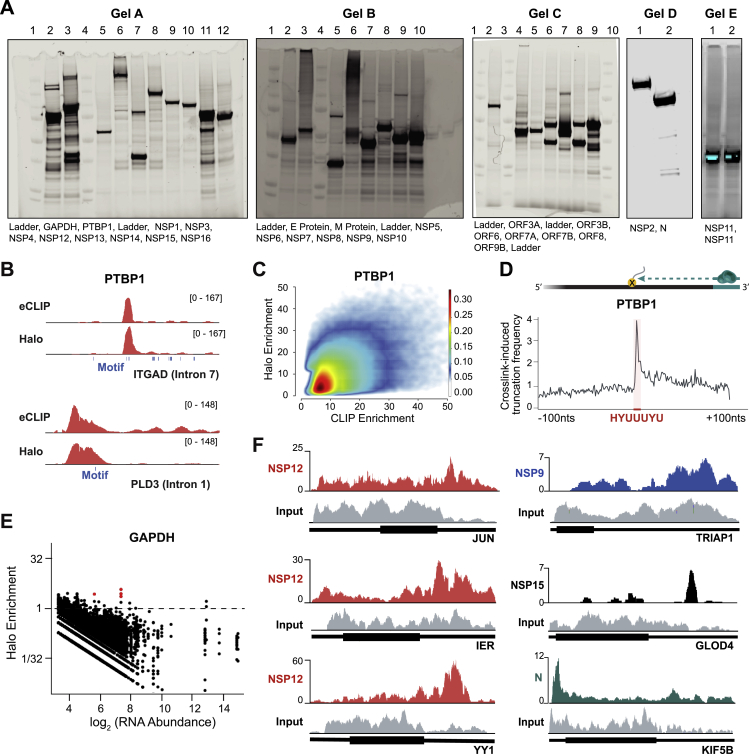
Global RNA Binding Maps of SARS-CoV-2 Proteins, Related to Figure 1 (A) Protein expression gels of Halo-tagged SARS-CoV-2 proteins. Expression is visualized via AlexaFluor-660 conjugated Halo-ligand. (B) Example of eCLIP (top) and Halo (bottom) enrichments are plotted for PTBP1 over intronic regions of ITGAD mRNA. The location of the corresponding PTBP1 recognition motif (blue boxes) are shown. (C) Density scatterplot of the enrichment levels of PTBP1 over all human RNA regions as measured by eCLIP (x axis) compared to the enrichment levels as measured by Halo (y axis) for all RNAs identified as significantly enriched by eCLIP. (D) Cartoon illustrating protein-adduct mediated reverse transcriptase read stops at binding motifs (top). PTBP1 crosslink-induced truncation frequency relative to known PTBP1 motif (HYUUUYU, shown in red). (E) Scatterplot of RNA abundance (log scale, x axis) compared to Halo enrichment (log scale, y axis) for the GAPDH protein across all 100-nucleotide windows of all annotated human RNAs (exon and introns) are plotted. Windows with significant enrichment are shown in red. (F) Representative tracks illustrating different mRNA binding patterns in Halo captures of NSP12 (red), NPS9 (blue), NSP15 (black), and N-protein (blue). Input tracks are presented for each mRNA (gray).

**Figure 1:**
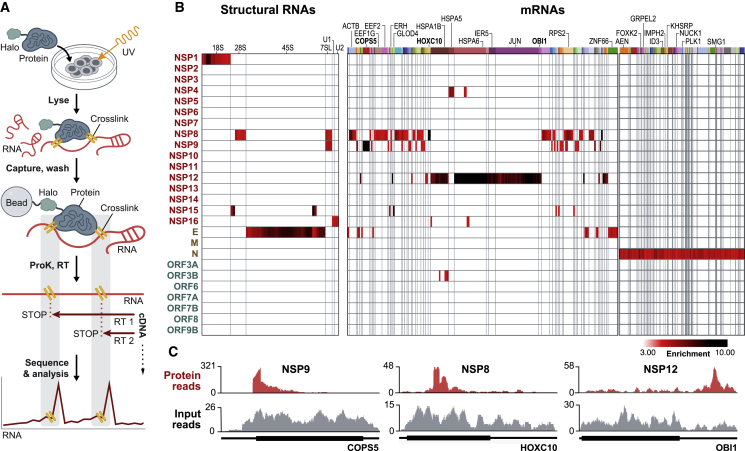
Global RNA Binding Maps of SARS-CoV-2 Proteins (A) Schematic of our approach. (B) Enrichment heatmap of each SARS-CoV-2 protein (rows) by significantly enriched 100-nt RNA bins (columns; p < 0.001 and enrichment > 3-fold; STAR Methods). Shared colored bars indicate multiple bins within the same mRNA. For spacing reasons, the 82 mRNAs bound by N protein are displayed separately. (C) Examples of sequencing reads over specific mRNAs for viral proteins (red) relative to input RNA coverage (gray) are shown. Coding regions (thick lines) and untranslated regions (thin lines) are shown for each mRNA. See also Table S1.

We cloned all 27 of the known SARS-CoV-2 viral proteins into mammalian expression vectors containing an N-terminal HaloTag (Los et al., 2008; Figure S1A; STAR Methods), expressed each in HEK293T cells, and exposed them to UV light to covalently crosslink proteins to their bound RNAs. We then lysed the cells and purified each viral protein using stringent denaturing conditions to disrupt any non-covalent associations and capture those with a UV-mediated interaction (Figure 1A; STAR Methods). As positive and negative controls, we purified a known human RNA binding protein (PTBP1) and a metabolic protein (GAPDH) (Figures S1A–S1E).

We successfully purified 26 of the 27 viral proteins (Figure S1A; full-length S was not soluble when expressed). We found that 10 viral proteins (NSP1, NSP4, NSP8, NSP9, NSP12, NSP15, NSP16, ORF3b, N, and E) bind to specific host RNAs (p < 0.001; Figure 1B; Table S1), including 6 structural ncRNAs and 142 mRNAs (Table S1). These include mRNAs involved in protein translation (e.g., COPS5, EIF1, and RPS12,), protein transport (ATP6V1G1, SLC25A6, and TOMM20), protein folding (HSPA5, HSPA6, and HSPA1B), transcriptional regulation (YY1, ID4, and IER5), and immune response (JUN, AEN, and RACK1) (false discovery rate [FDR] < 0.05; Figures 1B and S1F). Importantly, the observed interactions are highly specific for each viral protein, and each protein binds to a precise region within each RNA (Figures 1C and S1F).

Using these data, we identified several viral proteins that interact with structural ncRNA components of the spliceosome (U1 and U2 small nuclear RNA [snRNA]), the ribosome (18S and 28S rRNA), and the SRP (7SL) (Figure 1B). Because these molecular machines are essential for three essential steps of protein production—mRNA splicing, translation, and protein trafficking—we focused on their interactions with viral proteins to understand their functions and mechanisms in SARS-CoV-2 pathogenesis.

### NSP16 Binds to the Pre-mRNA Recognition Domains of the U1 and U2 snRNAs

After transcription in the nucleus, nascent pre-mRNAs are spliced to generate mature mRNAs that are translated into protein. Splicing is mediated by a complex of ncRNAs and proteins known as the spliceosome. Specifically, the U1 snRNA hybridizes to the 5′ splice site at the exon-intron junction, and the U2 snRNA hybridizes to the branchpoint site in the intron to initiate splicing of virtually all human mRNAs (Séraphin et al., 1988).

**Figure 2:**
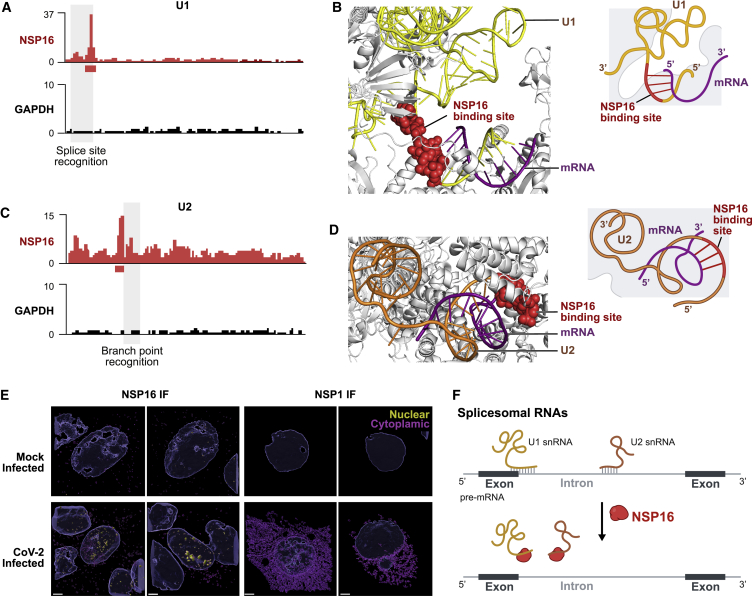
NSP16 Binds to U1 and U2 at Their mRNA Recognition Sites (A) NSP16 enrichment of reverse transcription stop positions across each nucleotide of U1 (red) compared with a control protein (GAPDH, black). The red box (below the x axis) represents most enriched nucleotide positions (U1, 9–13 nt). The gray-shaded box (overlay) outlines the position of the splice site recognition sequence. (B) Left: structure of the pre-catalytic human spliceosome (PDB: 6QX9; Charenton et al., 2019), highlighting the location of NSP16 binding site (red spheres) relative to U1 (yellow ribbon) and mRNA (purple ribbon). Right: schematic of the structure. (C) Enrichment across each nucleotide of U2 for NSP16 (red) and GAPDH (black). The red box demarcates most enriched nucleotide positions (U2, 27–34 nt). The gray-shaded box outlines the location of the branchpoint recognition sequence. (D) Structure of the pre-catalytic human spliceosome (PDB: 6QX9; Charenton et al., 2019) displaying the NSP16 binding site (red spheres), U2 (orange), and mRNA (purple). (E) Mock-infected (top) or SARS-CoV-2 infected (bottom) Vero E6 cells immunostained with a polyclonal antibody to NSP16 (left) or NSP1 (right). Imaris 3D reconstruction of the DAPI (nucleus) and NSP16 or NSP1 signal are shown for each protein. The signal contained within the 3D nuclear volume (blue) is shown in yellow and the cytoplasmic signal in purple. Scale bars, 3 μm. (F) Model: NSP16 binding to U1/U2 can affect mRNA recognition during splicing.

**Figure S2:**
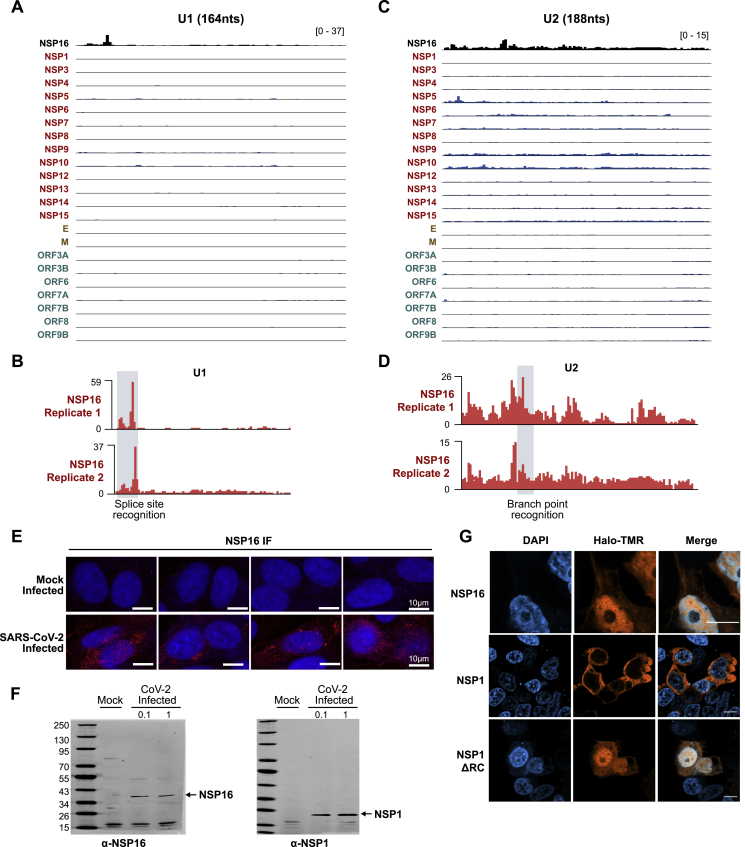
NSP16 Binds to the U1 and U2 Components of the Spliceosome at Their mRNA Recognition Sites, Related to Figure 2 (A) Comparison of U1 RNA enrichment across SARS-CoV-2 Halo capture datasets. (B) NSP16 binding traces along U1 RNA between two separate captures. Splice site recognition domain is highlighted in gray. (C) Comparison of U2 RNA enrichment across SARS-CoV-2 Halo capture datasets. (D) NSP16 binding traces along U2 RNA between two separate captures. Branch point recognition domain is highlighted in gray. (E) NSP16 immunofluorescence in Vero E6 cells infected (or mock infected) with SARS-CoV-2 at an MOI of 0.1 for 48h. Four representative fields are displayed, with size bar indicating 10 microns. (F) Western blot confirmation of NSP16 and NSP1 antibodies used to generate images in (E). Vero cells were infected (or mock infected) with SARS-CoV-2 at an MOI of 0.1 or 1; 72 hpi cells were lysed and probed by western blot with antibodies raised against NSP1 or NSP16. (G) Imaging of HEK293T cells transfected with Halo-tagged NSP16, NSP1, and NSP1ΔRC plasmids. Proteins are visualized using TMR-conjugated Halo-ligand (orange) and counter-stained with DAPI (blue). Scale bars indicate 10 microns.

We identified a highly specific interaction between the NSP16 viral protein and the U1 and U2 snRNAs (Figure 1B). Because U1 and U2 are small RNAs (164 and 188 nt, respectively), we noticed strong enrichment of NSP16-associated reads across the entire length of each. To more precisely define the binding sites, we exploited the well-described tendency of reverse transcriptase to preferentially terminate when it encounters a UV-crosslinked protein on RNA (König et al., 2010; Figures 1A and S1D). We determined that NSP16 binds to the 5′ splice site recognition sequence of U1 (Figures 2A, 2B, S2A, and S2B) and the branchpoint recognition site of U2 (Figures 2C, 2D, S2C, and S2D). These binding sites are highly specific to NSP16 relative to all of the other viral and human proteins (Figures 1B, S2A, and 2C). Consistent with its interaction with U1/U2, we observed that NSP16 localizes in the nucleus upon SARS-CoV-2 infection (Figures 2E, S2E, and S2F) and when expressed in human cells (Figure S2G).

### NSP16 Disrupts Global mRNA Splicing upon SARS-CoV-2 Infection

**Figure 3:**
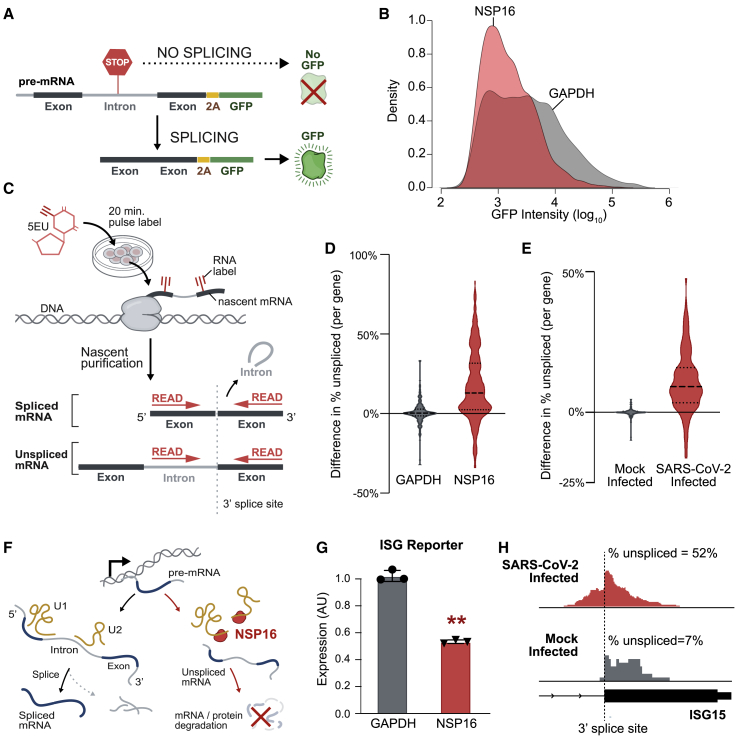
NSP16 Suppresses Host mRNA Splicing (A) Schematic of fluorescence reporter used to assay mRNA splicing. (B) GFP density plot of HEK293T cells expressing the GFP splicing reporter and either GAPDH (gray) or NSP16 (red). (C) Schematic of the nascent RNA purification method. (D) The percentage of unspliced difference for each gene between HEK293T cells transfected with GAPDH (gray) or NSP16 (red). The plot represents the merge of four independent biological replicates; replicates are plotted in Figure S4C. (E) Violin plot for SARS-CoV-2 infected human lung epithelial cells (MOI = 0.01, 48 h) compared with mock infection. Plots are merges of two biological replicates; replicates are plotted in Figure S4E. (F) Model. NSP16 binding to U1 and U2 can reduce overall mRNA and protein levels. (G) Expression of an IFN-stimulated gene (ISG) reporter upon transfection with GAPDH (gray) or NSP16 (red) after stimulation with IFN-β. Three independent biological replicates; ^∗∗^p < 0.01. (H) Example of nascent RNA sequencing at the intron of ISG15 (intron, line; exon, box) upon SARS-CoV-2 (red) or mock (gray) infection.

**Figure S3:**
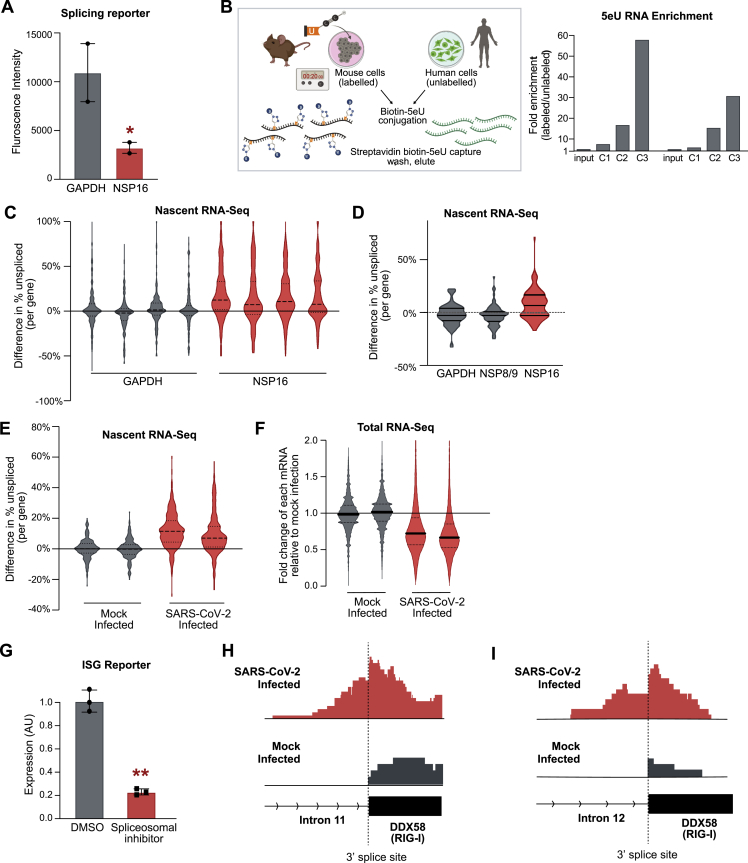
NSP16 Suppresses Host mRNA Splicing, Related to Figure 3 (A) Median of raw GFP fluorescence measured in splicing reporter assay performed in HEK293T cells expressing either Halo-GAPDH (gray) or Halo-NSP16 (red). Two independent biological replicates per condition. (B) Overview of nascent RNA-sequencing method, including 5eU nucleotide feeding, biotin click chemistry conjugation, and biotin-streptavidin-based iterative capture methods. Human/mouse mixing experimental data illustrates selective enrichment of labeled material over unlabeled material after three sequential captures. (C1 = capture 1 enrichment, C2 = capture 2 enrichment, etc.) (C) Violin plot depicting difference in unspliced reads per gene (defined as the difference between number of unspliced fragment divided by total fragments spanning the 3′ splice site between condition and median of all control samples) for HEK293T cells transfected with either GAPDH (gray) or NSP16 (red) for 48hrs. All four individual replicates are presented. (D) Violin plot depicting difference in unspliced reads per gene (relative to median of GAPDH) for HEK293T cells transfected with either GAPDH, NSP9, or NSP16 (red) for 48hrs. (E) Violin plot depicting difference in unspliced reads per gene (relative to median of the mock condition) for Calu3 cells infected with SARS-CoV-2 virus at an MOI of 0.01 for 48 hr (red) or uninfected (gray). Biological replicates are presented. (F) Violin plot depicting fold change in total steady-state mRNA levels (mRNA initially normalized to ncRNA and ratio is fold normalized mock treatment) for SARS-CoV-2 infected (red) compared to mock infected (gray) samples. Data is presented for two biological replicates for each condition. (G) Normalized expression of an interferon signaling reporter upon stimulation with IFN-β and treatment with madrasin spliceosomal inhibitor (red) or DMSO vehicle (gray). Three independent biological replicates were measured for each condition. (H-I) Representative nascent RNA tracks from SARS-Cov-2 infected (red) and mock-treated cells (gray) along Intron 11 and Intron 12 of interferon stimulated gene, RIG-I.

Based on the locations of the NSP16 binding sites relative to the mRNA recognition domains of the U1/U2 spliceosomal components, we hypothesized that NSP16 might disrupt splicing of newly transcribed genes (Figure 2F). To test this, we co-expressed NSP16 in human cells along with a splicing reporter derived from IRF7 (an exon-intron-exon minigene) fused to GFP (Majumdar et al., 2018). In this system, if the reporter is spliced, then GFP is made; if not, then translation is terminated (via a stop codon present in the first intron), and GFP is not produced (Figure 3A). We observed a more than 3-fold reduction in GFP levels in the presence of NSP16 compared with a control human protein (Figures 3B and S3A).

To explore whether NSP16 has a global effect on splicing of endogenous mRNAs, we measured the splicing ratio of each gene using nascent RNA sequencing. Specifically, we metabolically labeled nascent RNA by feeding cells for 20 min with 5-ethynyl uridine (5EU), purified and sequenced 5EU-labeled RNA, and quantified the proportion of unspliced fragments spanning the 3′ splice site of each gene (Figures 3C and S3B). We observed a global increase in the fraction of unspliced genes in the presence of NSP16 compared with controls (Figures 3D, S3C, and S3D).

Given that NSP16 is sufficient to suppress global mRNA splicing, we expect that its expression in SARS-CoV-2-infected cells would result in a global mRNA splicing deficit. To test this, we infected human lung epithelial cells (Calu3) with SARS-CoV-2 and measured the splicing levels of newly transcribed mRNAs compared with a mock-infected control. As expected, we observed a global increase in the fraction of unspliced transcripts upon SARS-CoV-2 infection, with ∼90% of measured genes showing increased intron retention (Figures 3E and S3E).

These results indicate that NSP16 binds to the splice site and branchpoint sites of U1/U2 to suppress global mRNA splicing in SARS-CoV-2-infected cells (Figure 3F). Although NSP16 is known to act as an enzyme that deposits 2′-O-methyl modifications on viral RNAs (Decroly et al., 2011), our results demonstrate that it also acts as a host virulence factor. Global disruption of mRNA splicing may act to decrease host protein and mRNA levels by triggering nonsense-mediated decay of improperly spliced mRNAs (Kurosaki et al., 2019). Consistent with this, we observed a strong global decrease in steady-state mRNA levels (relative to ncRNA levels) upon SARS-CoV-2 infection (Figure S3F).

### Inhibition of mRNA Splicing Suppresses the Host IFN Response to Viral Infection

Because many of the key genes stimulated by IFN are spliced, we reasoned that mRNA splicing would be critical for a robust IFN response. To test this, we utilized a reporter line engineered to express alkaline phosphatase upon IFN signaling (mimicking an antiviral response gene). This IFN-stimulated gene (ISG) reporter line can be stimulated using IFN-β and assayed for reporter induction. We observed strong repression of this IFN-responsive gene upon expression of NSP16 (Figure 3G) and upon addition of a small molecule that interferes with spliceosomal assembly (Figure S3G). These results demonstrate that one outcome of NSP16-mediated inhibition of mRNA splicing is to reduce the host cells’ innate immune response to virus recognition. Consistent with such a role, we observed an increase in intron retention in multiple IFN-responsive genes (such as ISG15 and RIG-I) upon SARS-CoV-2 infection (Figures 3H, S3H, and S3I).

### NSP1 Binds to 18S Ribosomal RNA in the mRNA Entry Channel of the 40S Subunit

**Figure S4:**
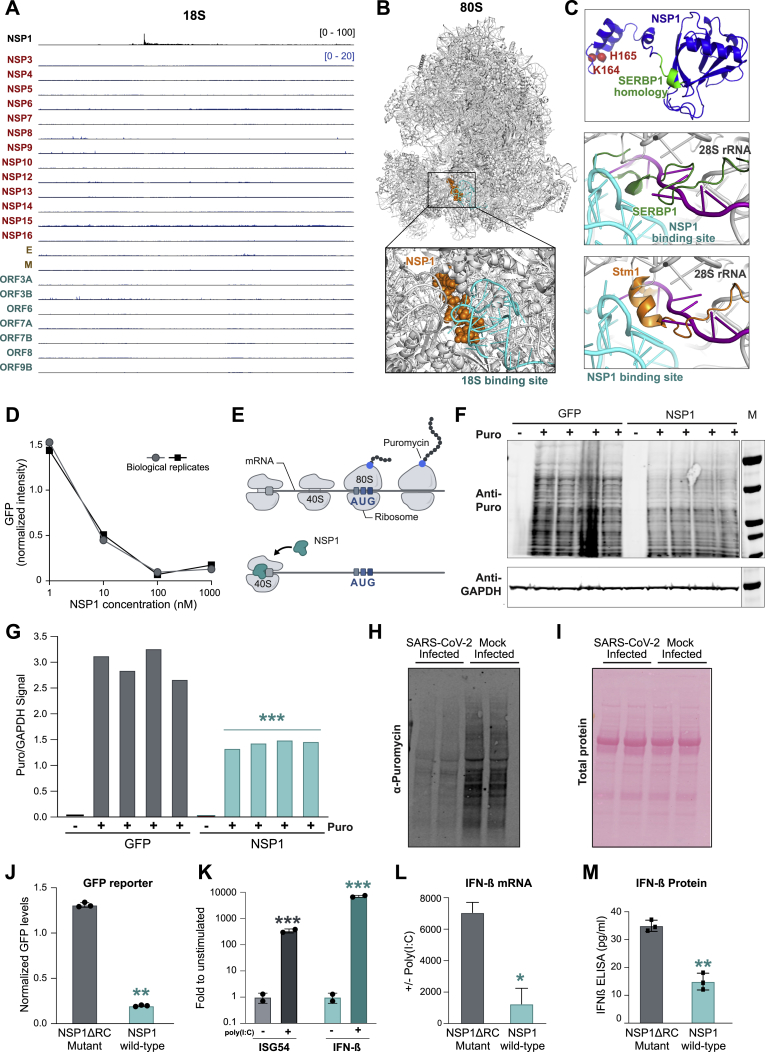
NSP1 Binds to the 18S Ribosomal RNA Near the mRNA Entry Channel to Suppress Global mRNA Translation in Cells, Related to Figure 4 (A) Comparison of 18S RNA enrichment across SARS-CoV-2 Halo capture datasets. (B) The location of NSP1 binding (orange spheres) relative to 18S binding site (cyan) upon known structure of the 80S ribosome (gray). (C) Predicted structure of NSP1 based on Robetta modeling. The critical C-terminal amino acids required for binding 18S (K164 and H165) are indicated as red spheres. The region of homology with SERBP1 is shown in green. The observed NSP1 binding sites on the 18S rRNA are demarcated in cyan on the structure of the human 40S ribosome (PDB: 6G5H; gray)(Ameismeier et al., 2018), relative to the mRNA path (purple; 6YAL)(Simonetti et al., 2020), and known clogging factors (E) SERBP1 (green; 6MTE)(Brown et al., 2018) and (F) Stm1 (orange; 4V88)(Ben-Shem et al., 2011). (D) An mRNA encoding GFP was added to HeLa cell extracts along with different concentrations of purified NSP1 protein (x axis). The amount of GFP protein measured relative to the median of replicates for a buffer only control is shown (y axis). Two independent dose titrations were performed and are shown on top of each other. (E) Schematic illustrating puromycin tagging of newly translated proteins via the SuNSET method. If the level of ongoing translation is high, we expect to detect a large amount of newly generated proteins containing puromycin; if global translation is suppressed, we expect to observe a decrease in the amount of puromycin integrated into proteins. (F) Western blot of global puromycin incorporation into proteins of HEK293T cells transfected with either Halo-GFP (left) or Halo-NSP1 (right). GAPDH levels were measured in the same lysates to normalize for total protein in the sample (bottom). (-) puro lanes indicate transfected samples that were not treated with puromycin. (G) Quantification of puromycin intensity across each lane of the gel in Panel F. The ratio of puromycin signal over total GAPDH signal is plotted for individual replicates. (H) Vero E6 cells were infected (or mock infected) with SARS-CoV-2 at an MOI of 0.01. 48hpi cells were labeled with media containing puromycin, and lysates were probed by western blot. (I) As a control for total protein levels, after samples in (G) were run on a SDS-PAGE gel, transferred to nitrocellulose, and total proteins were stained with PONCEAU before blocking/antibody detection of puromycin signal. (J) Normalized GFP fluorescence intensity of GFP reporter co-transfected in HEK293T cells in the presence of the NSP1ΔRC mutant that does not bind to 18S (gray) or NSP1 (cyan) proteins. Three independent biological replicates were measured for each sample. Note: This experiment was performed alongside the various controls displayed in Figure 4D and are plotted on the same scale. (K) mRNA levels of ISG54 and IFN-β following stimulation with poly(I:C) normalized to levels in unstimulated A549 cells. (L and M) mRNA and protein levels of IFN-β following stimulation with poly(I:C) normalized to levels in unstimulated A549 cells transfected with NSP1ΔRC mutant (gray) or NSP1 (cyan). Two independent biological replicates were measured for each condition. In all panels, error bars represent standard deviation across replicates, and dots represent individual values for each replicate. ^∗^ indicates p < 0.05, ^∗∗^p < 0.01, and ^∗∗∗^p < 0.001.

When exported to the cytoplasm, spliced mRNA is translated into protein on the ribosome. Initiation of translation begins with recognition of the 5′ cap by the small 40S subunit (which scans the mRNA to find the first start codon). We observed that NSP1 binds exclusively to the 18S ribosomal RNA (Figures 1B and S4A)—the structural RNA component of the 40S ribosomal subunit.

Several roles of NSP1 have been reported in SARS-CoV and MERS-CoV, including roles in viral replication, translational inhibition, transcriptional inhibition, mRNA degradation, and cell cycle arrest (Brockway and Denison, 2005; Kamitani et al., 2009; Lokugamage et al., 2015; Narayanan et al., 2015). One of the reported roles of NSP1 in SARS-CoV is that it can associate with the 40S ribosome to inhibit host mRNA translation (Kamitani et al., 2009; Tanaka et al., 2012), but it remains unknown whether this association is due to interaction with the ribosomal RNA, protein components of the ribosome, or other auxiliary ribosomal factors. Accordingly, the mechanisms by which NSP1 acts to suppress protein production remain elusive.

**Figure 4:**
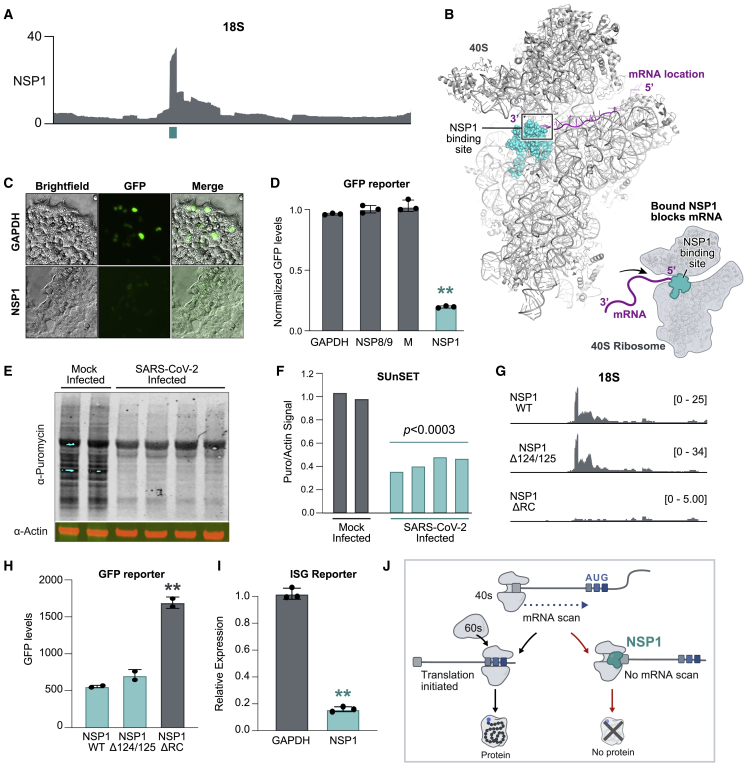
NSP1 Binds to 18S Near the mRNA Entry Channel to Suppress Translation (A) NSP1 enrichment across each nucleotide of 18S. The cyan box indicates the most enriched nucleotides of NSP1 binding (18S, 607–644 nt). (B) The location of NSP1 binding (cyan spheres) relative to the known structure of 40S (gray) and mRNA (purple ribbon). Right: schematic illustrating structure (Ameismeier et al., 2018) and how NSP1 binding would block mRNA entry. (C) Images of HEK293T cells co-expressing the GFP reporter and GAPDH (top) or NSP1 (bottom). (D) Flow cytometry quantification (mean intensity) of GFP in the presence of GAPDH, NSP8/9, M, or NSP1 proteins. Three independent biological replicates per condition. (E) Puromycin incorporation (top) or total actin levels (bottom) measured in Calu3 cells infected with SARS-CoV-2 (MOI = 0.01, 48 h) or a mock-infected control (left 2 lanes). (F) The ratio of puromycin signal over total actin signal is plotted for each individual replicate. (G) Read enrichment on 18S for an independent replicate of NSP1 wild type, NSP1 R124A/K125A mutant, and NSP1 K164A/H165A (ΔRC) mutant. (H) Flow cytometry analysis of HEK293T cells transfected with GFP and NSP1ΔRC mutant (gray), wild-type NSP1, or NSP1 R124A/K125A (cyan). (I) Quantification of the IFN-β response in the presence of GAPDH (gray) or NSP1 (cyan). (J) Schematic of how NSP1 acts to suppress mRNA translation. Error bars represent standard deviation across biological replicates, and dots represent individual values for each replicate; ^∗^p < 0.05 and ^∗∗^p < 0.01.

We mapped the location of NSP1 binding to a 37-nt region corresponding to helix 18 (Figure 4A), adjacent to the mRNA entry channel (Simonetti et al., 2020; Figure 4B). The interaction would position NSP1 to disrupt 40S mRNA scanning and prevent translation initiation (Figure 4B) and disrupt tRNA recruitment to the 80S ribosome and block protein production (Figure S4B). Interestingly, the NSP1 binding site includes the highly conserved G626 nucleotide, which monitors the minor groove of the codon-anticodon helix for tRNA binding fidelity (Ogle et al., 2001). We noticed that the C-terminal region of NSP1 has structural regions similar to SERBP1 (Brown et al., 2018) and Stm1 (Ben-Shem et al., 2011), two known ribosome inhibitors that bind in the mRNA entry channel to preclude mRNA access (Figure S4C). Consistent with this, a recent cryo-EM structure confirms that NSP1 binds to these same nucleotides of 18S within the mRNA entry channel (Thoms et al., 2020).

### NSP1 Suppresses Global Translation of Host mRNAs upon SARS-CoV-2 Infection

Given the location of NSP1 binding on the 40S ribosome, we hypothesized that it could suppress global initiation of mRNA translation. To test this, we performed *in vitro* translation assays of a GFP reporter in HeLa cell lysates and found that addition of NSP1 led to potent inhibition of translation (Figure S4D). We observed a similar NSP1-mediated translational repression when we co-expressed NSP1 and a GFP reporter gene in HEK293T cells (Figures 4C and 4D). In contrast, we did not observe this inhibition when we expressed other SARS-CoV-2 proteins (NSP8, NSP9, or M) or human proteins (GAPDH) (Figure 4D).

To determine whether NSP1 leads to translational inhibition of endogenous proteins in human cells, we used a technique called surface sensing of translation (SUnSET) to measure global protein production levels (Schmidt et al., 2009). In this assay, translational activity is measured by the level of puromycin incorporation into elongating polypeptides (Figure S4E). We observed a strong reduction in the level of global puromycin integration in cells expressing NSP1 compared with cells expressing GFP (Figures S4F and S4G).

Because NSP1 expression is sufficient to suppress global mRNA translation in human cells, we hypothesized that SARS-CoV-2 infection would also suppress global translation. To test this, we infected a human lung epithelial (Calu3) or monkey kidney (Vero) cell line with SARS-CoV-2 and measured nascent protein synthesis levels using SUnSET. We observed a strong reduction of global puromycin integration upon SARS-CoV-2 infection in both cell types (Figures 4E, 4F, S4H, and S4I).

To explore whether NSP1 binding to 18S rRNA is critical for translational repression, we generated a mutant NSP1 in which two positively charged amino acids (K164 and H165) in the C-terminal domain were replaced with alanine residues (Figure S4C; Narayanan et al., 2008). We observed complete loss of *in vivo* contacts with 18S (Figure 4G); because this mutant disrupts ribosome contact, we refer to it as NSP1ΔRC. We co-expressed GFP and NSP1ΔRC in HEK293T cells and found that the mutant fails to inhibit translation (Figures 4H and S4J). In contrast, mutations to the positively charged amino acids at positions 124/125 do not affect 18S binding (Figure 4G) or the ability to inhibit translation (Figure 4H).

These results demonstrate that NSP1 binds in the mRNA entry channel of the ribosome and that this interaction is required for translational inhibition of host mRNAs upon SARS-CoV-2 infection.

### NSP1-Mediated Translational Inhibition Suppresses the Host IFN Response

We explored whether NSP1 binding to 18S rRNA suppresses the ability of cells to respond to IFN-β stimulation upon viral infection. We transfected ISG reporter cells with NSP1, stimulated with IFN-β, and observed robust repression of the IFN-responsive gene (>6-fold; Figure 4I). To confirm that this NSP1-mediated repression occurs in human cells upon activation of double-stranded RNA (dsRNA)-sensing pathways typically triggered by viral infection, we treated a human lung epithelial cell line (A549) with poly(I:C), a molecule that is structurally similar to dsRNA and known to induce an antiviral innate immune response (Alexopoulou et al., 2001; Kato et al., 2006) (Figure S4K). We observed marked downregulation of IFN-β protein and endogenous IFN-β-responsive mRNAs in the presence of NSP1 but not in the presence of NSP1ΔRC (Figures S4L and S4M). These results demonstrate that NSP1, through its interaction with 18S rRNA, suppresses the innate immune response to virus recognition (Figure 4J).

### The Viral 5′ Leader Protects mRNA from NSP1-Mediated Translational Inhibition

**Figure S5:**
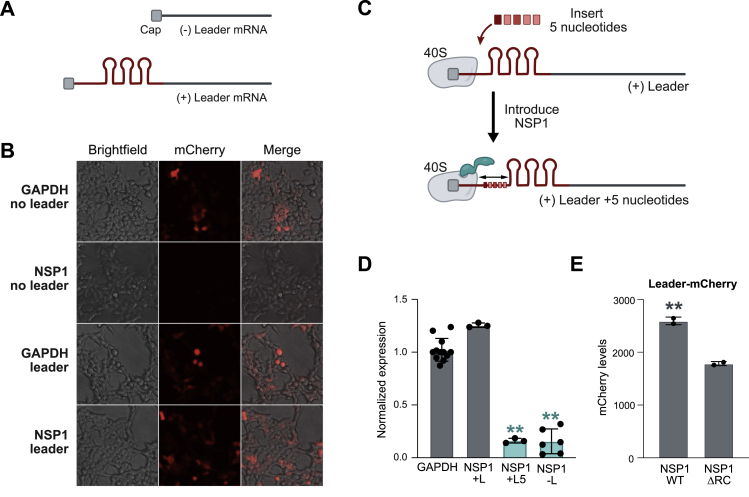
The 5′ Viral Leader Sequence Protects mRNAs from NSP1-Mediated Translational Inhibition, Related to Figure 5 (A) A schematic of the experimental design containing two reporter RNAs encoding fluorescent proteins, without the viral leader (top) and with the viral leader sequence appended to the 5′ end of the mRNA (bottom). Viral leader represented by three stem-loops in red. (B) Representative images of HEK293T cells co-transfected with GAPDH or NSP1 along with mCherry RNA with or without SARS-CoV-2 leader sequence. (C) Schematic illustrating the insertion of 5 nucleotides between the 5′ cap and the viral leader sequence. NSP1 protein represented in red. (D) Quantification of mCherry expression in HEK293T cells transfected with mCherry RNAs, fused to different 5′ leader variants, and either GAPDH or NSP1. Values are normalized to the median values of mCherry levels from control condition (GAPDH with + mCherry). At least 3 independent biological replicates per condition. Dots represent value for each independent replicate (e.g., NSP1 -L contains 6 independent replicates). (E) Quantification of mCherry expression from HEK293T cells transfected with Halo-tagged NSP1 WT or NSP1ΔRC mutant, along with leader-mCherry expressing plasmids. Values are normalized to the median values of mCherry levels in control sample (NSP1 with + leader-mCherry). Two independent biological replicates were measured per condition. In all panels, error bars represent standard deviation across replicates, and dots represent individual values for each replicate. ^∗^ indicates p < 0.05 and ^∗∗^p < 0.01.

**Figure 5:**
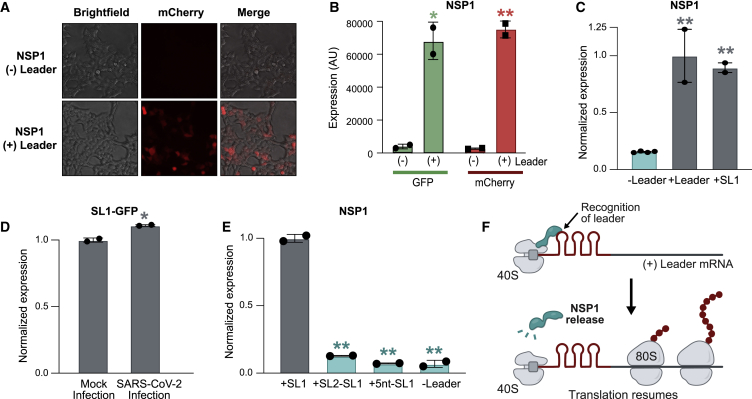
The 5′ Viral Leader Protects mRNA from NSP1-Mediated Translational Inhibition (A) Images of cells co-transfected with NSP1 and mCherry alone (− leader, top) or mCherry fused to the SARS-CoV-2 leader (+ leader, bottom). (B) GFP (green) or mCherry (red) levels when fused to the viral leader (+ leader, right) or lacking the viral leader (− leader, left). (C) GFP reporter with no leader (left), full leader (center), or stem loop 1 (SL1) upon NSP1 expression. (D) Calu3 cells expressing SL1 fused to GFP. Cells were mock or SARS-CoV-2 infected (MOI = 0.1), and GFP expression was measured 24 h after infection by flow cytometry. (E) GFP reporter containing SL1 (left), a swap of SL2 and SL1 (SL2-SL1), insertion of 5 nt between the 5′ end and SL1 (+5 nt-SL1), or no leader. GFP protein level was measured for each condition upon expression of NSP1. (F) Proposed model of how NSP1 binding to the viral leader can allosterically modulate NSP1 structure to protect mRNAs in *cis*. Error bars represent standard deviation across biological replicates, and dots represent individual replicate values; ^∗^p < 0.05 and ^∗∗^p < 0.01.

Because NSP1 blocking the mRNA entry channel would affect host and viral mRNA translation, we explored how translation of viral mRNAs is protected from NSP1-mediated translational inhibition. Many viruses contain 5′ untranslated regions that regulate viral gene expression and translation (Gaglia et al., 2012); all SARS-CoV-2-encoded subgenomic RNAs contain a common 5′ leader sequence that is added during negative-strand synthesis (Kim et al., 2020b). We explored whether the leader sequence protects viral mRNAs from translational inhibition by fusing the viral leader sequence to the 5′ end of GFP or mCherry reporter genes (Figure S5A). We found that NSP1 fails to suppress translation of these leader-containing mRNAs (Figures 5A, 5B, and S5B). We dissected the leader sequence and found that the first stem loop (SL1) is sufficient to prevent translational suppression upon NSP1 expression (Figure 5C) or SARS-CoV-2 infection (Figure 5D).

We considered three models for how the leader could protect viral mRNAs: (1) it could compete with the ribosome for NSP1 binding, (2) it could directly recruit free ribosomes, or (3) NSP1 could bind to the leader independent of its ribosome interaction to allosterically modulate the NSP1-ribosome interaction. We reasoned that if the leader competes for NSP1 binding or directly recruits free ribosomes, then the presence of SL1 should be sufficient for protection, regardless of its precise position in the 5′ UTR. In contrast, if the leader allosterically modulates ribosome binding, then the spacing between the 5′ cap (which is bound to NSP1-40S) and SL1 would be critical for protection. To distinguish between these models, we swapped the location of SL1 and SL2 in the 5′ leader or inserted 5 nt between the 5′ cap and SL1 (Figure S5C) and found that both mutants ablate protection (Figures 5E and S5D).

These results indicate that an mRNA requires the 5′ leader to be precisely positioned relative to the NSP1-bound 40S ribosome to enable translational initiation (Figure 5F). Although many aspects of this allosteric model remain to be explored, it would explain how leader-mediated protection can occur on an mRNA only when present in *cis*. Moreover, this model suggests that NSP1 might also act to further increase viral mRNA translation by actively recruiting the ribosome to its own mRNAs. Consistent with this, we observed a consistent, ∼20% increase in translation of leader-containing reporter levels upon viral infection (Figure 5D) or expression of NSP1 (Figure S5E).

### NSP8 and NSP9 Bind to the 7SL RNA Component of SRP

Upon engaging the start codon in an mRNA, the 60S subunit of the ribosome is recruited to form the 80S ribosome, which translates mRNA. SRP is a universally conserved complex that binds to the 80S ribosome and acts to co-translationally scan the nascent peptide to identify hydrophobic signal peptides present in integral membrane proteins and proteins secreted from the plasma membrane (Akopian et al., 2013). When these are identified, SRP triggers ribosome translocation to the endoplasmic reticulum (ER) to ensure proper folding and trafficking of these proteins to the cell membrane (Akopian et al., 2013).

**Figure 6:**
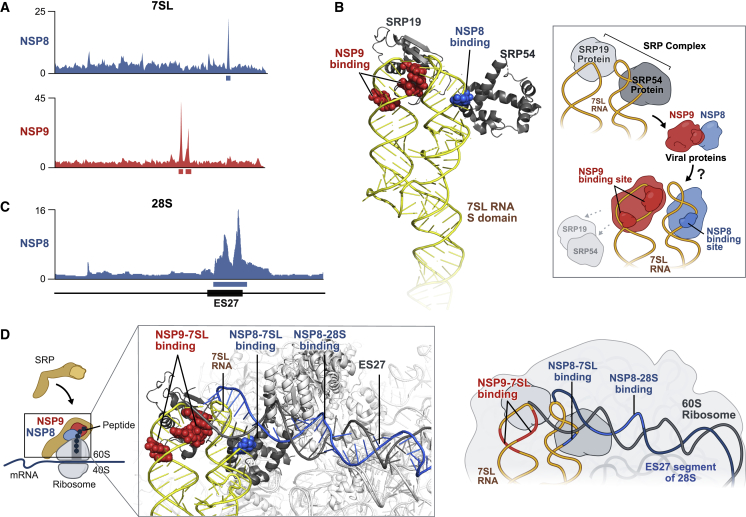
NSP8 and NSP9 Bind to 7SL RNA of the SRP (A) Enrichment of reverse transcription stop positions across each nucleotide of 7SL is shown for NSP8 (blue) and NSP9 (red). Red (7SL, 142–143 nt; 7SL, 149–151 nt) and blue (7SL, 193–194 nt) boxes demarcate the most enriched nucleotide positions. (B) The locations of the NSP8 (blue spheres) and NSP9 (red spheres) binding sites on the S domain of 7SL (yellow ribbon) structure relative to SRP54 and SRP19 (gray) (PDB: 1MFQ; Kuglstatter et al., 2002). Right: schematic of the structure and model of how NSP8/9 binding to 7SL could affect SRP protein binding. (C) Read enrichment across each nucleotide of 28S for NSP8 (blue). The black box indicates the location of the ES27 expansion sequence (28S, 2,889–3,551 nt). The blue box indicates the most enriched nucleotide position on 28S rRNA (28S, 3,017–3,529 nt). (D) The locations of the NSP8 (blue) and NSP9 (red) binding sites relative to the structure of the SRP-ribosome complex (PDB: 3JAJ; Voorhees and Hegde, 2015) superimposed on the structure of the ES27 region of 28S (Ebp1-ribosome complex; PDB: 6SXO; Wild et al., 2020). The observed NSP8 binding site in the ES27 region of 28S (gray) is demarcated in blue, and the NSP8 (blue) and NSP9 (red) binding sites on 7SL (yellow) are highlighted. Right: schematic illustrating the interaction between the ribosome and SRP.

**Figure S6:**
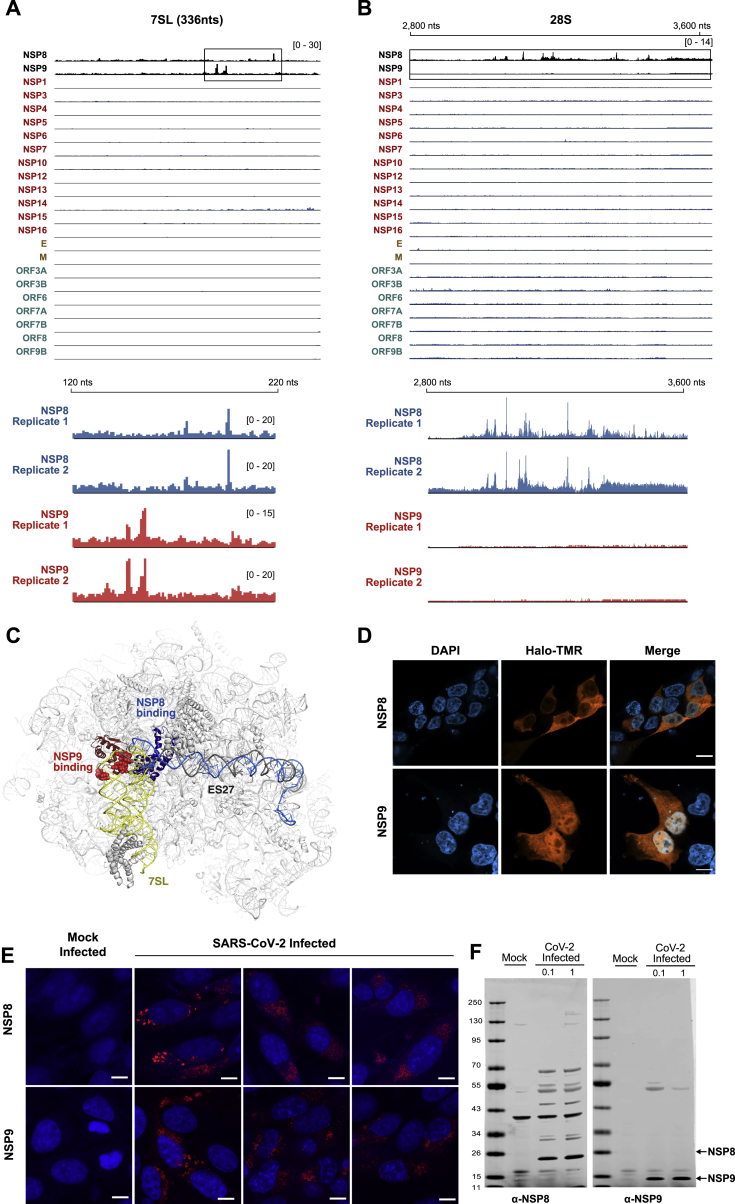
NSP8 and NSP9 Bind to the 7SL RNA Component of the SRP, Related to Figure 6 (A) Comparison of 7SL RNA second read enrichment across viral protein capture datasets (top) with region of highest enrichment for NSP8/9 boxed. Independent expression, purification, and sequencing experiments for NSP8 and NSP9 were performed and are shown. (B) Comparison of 28S RNA enrichment across SARS-CoV-2 Halo capture datasets (top). Replicate representative tracks of NSP8 (blue) and NSP9 (red) on 28S rRNA are presented below. (C) Full view of 80S ribosome structure, interfaced with SRP (7SL RNA, yellow line), NSP9 binding sites on 7SL (red circles), and NSP8 binding sites on 7SL (dark blue circles) and on ES27 expansion segment on the 28S ribosomal RNA (light blue line). (D) Imaging of HEK293T cells transfected with Halo-NSP8 or Halo-NSP9 plasmids. Proteins are visualized using TMR-conjugated Halo-ligand (orange) and counter-stained with DAPI (blue) nuclear stain. Size bars indicate 10 microns. (E) Vero E6 cells were infected (or mock infected) with SARS-CoV-2 at an MOI of 0.1 for 48h, before fixing and staining with an antibody raised against NSP8 or NSP9. Cells are counter-stained with DAPI. Size bars indicate 10 microns. (F) Western blot confirmation of NSP8 and NSP9 antibodies used to generate images in (E). Vero cells were infected (or mock infected) with SARS-CoV-2 at an MOI of 0.1 or 1; 72 hpi cells were lysed and probed by western blot with antibodies raised against NSP8 or NSP9.

We identified two viral proteins, NSP8 and NSP9, that bind at distinct and highly specific regions in the S domain of the 7SL RNA scaffold of SRP (Figures 6A and S6A). NSP8 interacts with 7SL in the region bound by SRP54 (the protein responsible for signal peptide recognition, SRP-receptor binding, and ribosome translocation; Akopian et al., 2013; Holtkamp et al., 2012; Figure 6B). NSP9 binds to 7SL in the region that is bound by the SRP19 protein (Figure 6B), which is required for proper folding and assembly of the SRP (including proper loading of SRP54; Akopian et al., 2013).

Because SRP scans nascent peptides co-translationally, we were intrigued to find that NSP8 also forms a highly specific interaction with 28S rRNA (the structural component of the 60S subunit) (Figures 6C and S6B). The binding site on 28S rRNA corresponds to the largest human-specific expansion segment in the ribosome, referred to as ES27 (Parker et al., 2018). ES27 is highly dynamic and, thus, has not been resolved in most ribosome structures (Zhang et al., 2014). However, when engaged by specific factors, ES27 can become ordered and has been shown recently to be capable of interacting with the ribosome exit tunnel adjacent to the 60S binding site of SRP (Figures 6D and S6C; Wild et al., 2020).

These observations suggest that NSP8 and NSP9 bind to the co-translational SRP complex. Consistent with this, we find that NSP8 and NSP9 localize broadly throughout the cytoplasm when expressed in human cells (Figure S6D) or upon SARS-CoV-2 infection (Figures S6E and S6F).

### NSP8 and NSP9 Suppress Protein Integration into the Cell Membrane

**Figure S7:**
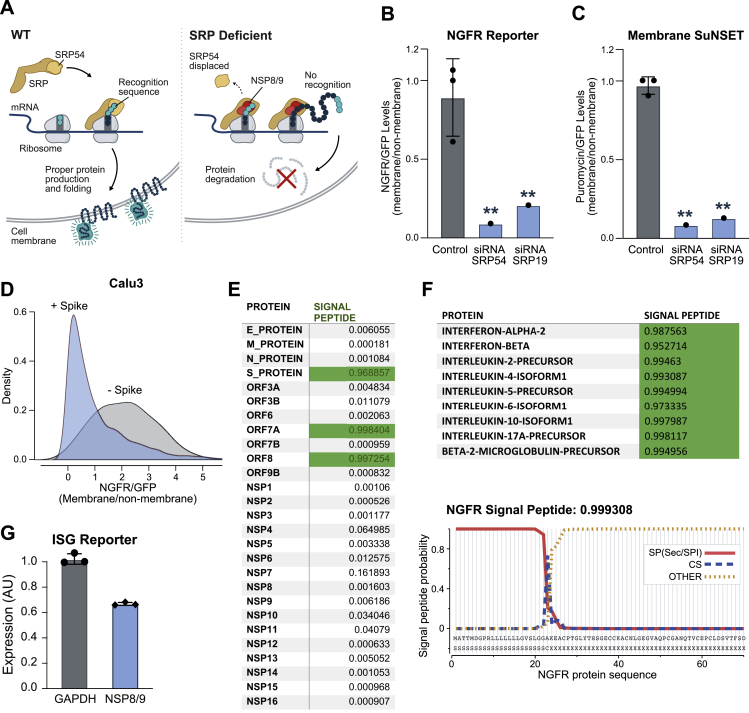
NSP8 and NSP9 Inhibit M and Secretory Protein Function, Related to Figure 7 (A) Schematic illustrating Signal Recognition Particle-mediated recognition and translocation of nascent membrane and secreted proteins (left). Upon SRP dysfunction, membrane and secreted proteins are predicted to be mislocalized and degraded (right). (B) Quantification of truncated Nerve Growth Factor Receptor (NGFR) fluorescence normalized to eGFP fluorescence (NGFR:GFP) from HEK293T cells transfected with control EED plasmid together with siRNAs targeting protein components of Signal Recognition Particle, SRP54 and SRP19. (C) Quantification of Membrane SuNSET puromycin staining fluorescence normalized to eGFP fluorescence (Puromycin:GFP) from HEK293T cells transfected with control EED plasmid together with with siRNAs targeting protein components of Signal Recognition Particle, SRP54 and SRP19. Three independent replicates for control and one replicate for siRNA treatments within this experiment. (D) NGFR:GFP ratio from Calu3 human lung epithelial cells infected with SARS-CoV-2 for 24 hr at an MOI of 0.1. Density comparison between Spike positive cells in virally infected condition to Spike negative cells in virally infected condition. (E) Signal P analysis of open reading frames of SARS-CoV-2 expressed proteins utilized in study. Proteins with greater than 0.95 predicted probability indicated Signal P algorithm are highlighted in green. (F) Top: Signal P analysis of open reading frames of various immunoregulatory cytokines and proteins, including Interferon Beta and Beta-2-Microglobulin-Precursor. Bottom: Signal P analysis of NGFR (membrane reporter) amino acid sequence and plot of signal peptide probability along the first 70 amino acids of NGFR sequence. In all panels, error bars represent standard deviation across replicates, and dots represent individual values for each replicate. ^∗^ indicates p < 0.05 and ^∗∗^p < 0.01. (G) Expression of an interferon stimulated gene reporter upon transfection with GAPDH or NSP8 and NSP9 (in combination), followed by stimulation with IFN-β. We note that because this assay measures intensity across a population of cells, any cells that are not transfected by NSP8/9 would not show this effect and would lead to a smaller overall difference than might occur within individual cells. In contrast, NGFR and SUNSET flow cytometry measurements (B-C) represent analysis of cells expressing NSP8/9.

Because NSP8 and NSP9 binding on 7SL are positioned to disrupt SRP function, we hypothesized that they may alter translocation of secreted and integral membrane proteins (Figure S7A).

**Figure 7:**
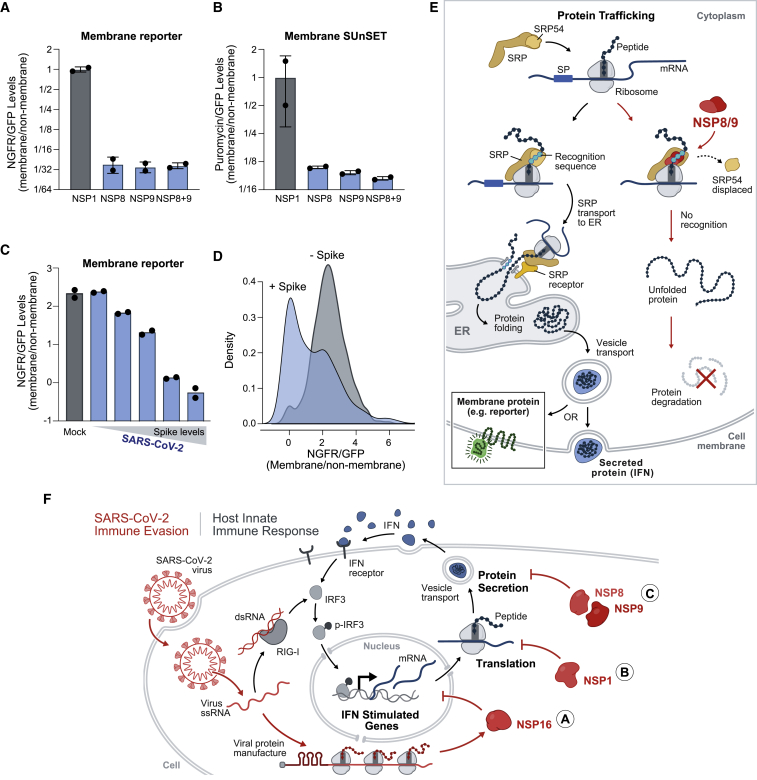
NSP8 and NSP9 Inhibit Membrane and Secretory Protein Trafficking (A) Quantification of HEK293T cells transfected with plasmids co-expressing GFP-tagged NSPs and the NGFR membrane protein. Plotted is the ratio of NGFR to GFP levels for each condition. (B) The ratio of puromycin-containing proteins at the cell membrane normalized to GFP expression for each condition. (C) Quantification of two mRNA reporters containing SL1 fused to GFP (leader-GFP) or NGFR (leader-NGFR) in Vero cells infected with SARS-CoV-2 or mock infected for 24 h (MOI, 0.1). Plotted is the ratio of leader-NGFR to leader-GFP, binned by increasing amounts of S protein. (D) Density plot for leader-NGFR to leader-GFP ratios in virally infected Vero cells or mock-treated controls. Replicate conditions were merged for display. (E) Model of how NSP8/9 act to suppress SRP-dependent protein trafficking upon viral infection. (F) A model of how SARS-CoV-2 suppresses host immune responses through multi-pronged inhibition of core cellular functions. Cellular mechanisms are shown in gray and viral mechanisms in red. Error bars represent standard deviation across independent biological replicates, and dots represent individual values for each replicate; ^∗^p < 0.05 and ^∗∗^p < 0.01.

To test this, we expressed an SRP-dependent membrane protein (nerve growth factor receptor [NGFR]; Izon et al., 2001) fused via an internal ribosome entry site (IRES) to a non-membrane GFP (Figure S7F). In this system, when a perturbation specifically affects membrane protein levels, we expect to see a decrease in the ratio of membrane to non-membrane protein levels. To ensure that the NGFR reporter accurately reports SRP function, we treated HEK293T cells with small interfering RNAs (siRNAs) against SRP54 or SRP19 and found that both lead to a dramatic reduction of the NGFR membrane protein relative to the non-membrane GFP protein (Figure S7B). Similarly, we found that expression of NSP8 and NSP9 (alone or together) led to a striking reduction in expression of NGFR relative to GFP (Figure 7A). Expression of control proteins did not specifically affect NGFR levels (Figures 7A and S7B).

To determine whether there is a global effect on membrane protein levels, we utilized the SUnSET method to measure puromycin levels in membrane proteins using flow cytometry (STAR Methods). We confirmed that disruption of SRP leads to a global reduction in puromycin levels in the cell membrane (Figure S7C). We observed a comparable global reduction of puromycin-labeled membrane proteins upon expression of NSP8 or NSP9 individually or together but not with control proteins (Figures 7B and S7C).

### SARS-CoV-2 Infection Suppresses Protein Integration into the Cell Membrane

Because NSP8 and NSP9 are each sufficient to suppress protein integration into the cell membrane, we anticipated that SARS-CoV-2 infection would lead to similar suppression. However, determining whether SARS-CoV-2 infection specifically affects membrane protein expression is confounded by the fact that NSP1 inhibits translation of membrane and non-membrane proteins upon infection.

To address this, we co-expressed a membrane protein reporter (NGFR) containing the 5′ viral leader along with a non-membrane GFP reporter containing the viral leader. Upon viral infection, we observed a strong reduction in membrane protein levels (Figure 7C) but no reduction in non-membrane GFP levels (Figure 5D). To ensure that these effects are specific to SARS-CoV-2-infected cells, we separated individual cells in the infected population into those expressing the viral S protein (S+) and those not expressing the protein (S−). We found that the shift in membrane protein levels only occurred in S+ cells (Figure 7D), whereas the S- population resembled the mock-infected samples (Figure 7C). We observed a strong relationship between the level of S protein, likely reflecting the amount of viral replication in each cell, and the level of membrane protein suppression (Figure 7C). We observed this membrane protein-specific decrease upon infection of human lung epithelial cells (Calu3; Figure S7D) and monkey kidney cells (Vero; Figures 7C and 7D).

These results demonstrate that NSP8 and NSP9 bind to 7SL to disrupt SRP function and suppress membrane protein trafficking in SARS-CoV-2-infected cells. Although NSP8 and NSP9 are thought to be components of the virus replication machinery (Sutton et al., 2004), our results indicate that they play an additional role as host virulence factors. Because viral membrane proteins also require trafficking to the ER, viral disruption of SRP might also negatively impact virus propagation, unless viral proteins are trafficked in an SRP-independent manner (Figure S7E) or if NSP8/9 selectively affects host (but not viral) protein trafficking.

### Viral Disruption of Protein Trafficking Suppresses the IFN Response

Next we explored how disruption of SRP might be advantageous for virus propagation. Because secretion of IFN and other cytokines is dependent on the SRP complex for secretion (Figure S7F), a central component of the IFN response is dependent on SRP. Accordingly, we hypothesized that NSP8/9-mediated viral suppression of SRP would act to suppress the IFN response upon infection. To test this, we co-expressed NSP8 and NSP9 and observed a significant reduction in the IFN response relative to a control protein (Figure S7G).

These results suggest that SARS-CoV-2-mediated suppression of SRP-dependent protein secretion enables suppression of host immune defenses (Figure 7E). Interestingly, many proteins involved in anti-viral immunity, including most cytokines and MHC class I, are membrane anchored or secreted and are known to use the SRP pathway for transport (Vermeire et al., 2014; Figure S7F), suggesting that there may be other effects of SRP pathway inhibition on SARS-CoV-2 pathogenesis.

## Discussion

We identified several pathogenic functions of SARS-CoV-2 in human cells, including global inhibition of host mRNA splicing, protein translation, and membrane protein trafficking, and described the molecular mechanisms by which the virus acts to disrupt these essential cell processes. Interestingly, all of the viral proteins involved (NSP1, NSP8, NSP9, and NSP16) are produced in the first stage of the virus life cycle prior to generation of dsRNA products during virus genome replication. Because dsRNA is detected by host immune sensors and triggers the type I IFN response, disruption of these cellular processes allows the virus to replicate its genome while minimizing the host innate immune response.

Disruption of these three non-overlapping steps of protein production may represent a multi-pronged mechanism that synergistically acts to suppress the host antiviral response (Figure 7F). Specifically, the IFN response is usually boosted more than 1,000-fold upon virus detection (through amplification and feedback; Figure S4K), but each individual mechanism affects IFN levels ∼5- to 10-fold. Accordingly, if each independent mechanism moderately affects IFN levels, the three together may be able to achieve dramatic suppression of IFN (10^3^ = 1,000-fold). This multi-pronged mechanism may explain the molecular basis of the potent suppression of IFN observed in patients with severe COVID-19.

IFN is emerging not only as a determinant of disease severity but also as a potential treatment option (Zhou et al., 2020). Our work identifies several therapeutic opportunities for boosting IFN levels upon SARS-CoV-2 infection. For example, disrupting the interaction between NSP1 and 18S rRNA could allow cells to detect and respond to viral infection. Because many small-molecule drugs target ribosomal RNAs (Liaud et al., 2019), it may be possible to develop drugs to block NSP1-18S and other interactions. Additionally, disrupting the 5′ viral leader may be a potent antivirus strategy because it is critical for translation of all viral proteins. Because SL1 is a structured RNA, it may be possible to design small molecules that specifically bind this structure to suppress viral protein production (Hermann, 2016).

Viral suppression of these cellular functions is not exclusive to the IFN response and also affects other spliced, translated, secreted, and membrane proteins. Many proteins involved in anti-viral immunity are spliced and/or membrane anchored or secreted; for example, MHC class I, critical for antigen presentation to CD8 T cells at the cell surface of infected cells (Hansen and Bouvier, 2009). By antagonizing membrane trafficking, SARS-CoV-2 may prevent viral antigens from being presented on MHC and allow infected cells to escape T cell recognition and clearance. In this way, interference with these essential cellular processes might further aid SARS-CoV-2 to evade the host immune response.

More generally, we expect that insights gained from the SARS-CoV-2 protein-RNA binding maps will be critical for exploring additional viral mechanisms. Specifically, we identified many other interactions, including highly specific interactions with mRNAs. For example, NSP12 binds to the JUN mRNA (Figure S1E), which encodes the critical immune transcription factor c-Jun, which is activated in response to multiple cytokines and immune signaling pathways (Weston and Davis, 2007). We also identified an interaction between NSP9 and the start codon of the mRNA that encodes COPS5 (Figure 1C), the enzymatic subunit of the COP9 signalosome complex, which regulates protein homeostasis (Cope and Deshaies, 2003), suggesting that it might disrupt its translation. Interestingly, COPS5 (also known as JAB1) is known to bind and stabilize c-Jun protein (Claret et al., 1996), and several viruses are known to disrupt this protein (Lungu et al., 2008; Oh et al., 2006; Tanaka et al., 2006). Although it remains unknown what, if any, role these interactions have in virus-infected cells, the specificity suggests that they may provide a selective advantage for virus propagation.

Our results demonstrate that global mapping of RNA binding by viral proteins could enable rapid characterization of mechanisms of emerging pathogenic RNA viruses.

### Limitations of Study

Several limitations of our current study will need to be explored in future work. (1) Our mapping experiments were performed with uninfected human cells expressing tagged viral proteins. Accordingly, it remains possible that our maps may not fully capture all of the interactions that occur when human cells are infected, such as interactions that occur with virus-induced RNAs, in specific viral compartments, or that require multiple viral proteins. (2) Although we characterized the functional and mechanistic roles of several viral proteins and structural ncRNAs, we did not explore the roles of viral protein interactions with mRNAs. (3) How the virus disrupts fundamental cellular processes while maintaining its own production is still largely undefined. Although we showed that the 5′ leader is sufficient to relieve translational inhibition by NSP1, we still do not fully understand how this protection occurs and, specifically, how NSP1 might interact with the viral leader or allosterically modulate ribosome binding. Similarly, viral membrane proteins are dependent on trafficking to the ER, and how NSP8/9 might selectively affect ER translocation of host but not viral proteins remains to be explored. (4) Although we showed that viral disruption of these essential cellular functions can suppress IFN, other roles of host cell shutdown in viral pathogenesis and in suppressing other aspects of antiviral immunity, including possible roles in adaptive immune responses, have not been explored.

## STAR★Methods

### Key Resources Table

**Table:** 

REAGENT or RESOURCE	SOURCE	IDENTIFIER
**Antibodies**		

Anti-NSP1 sheep polyclonal antibody	MRC-University of Glasgow Centre for Virus Research (CVR)	N/A
Anti-NSP8 sheep polyclonal antibody	MRC CVR	N/A
Anti-NSP9 sheep polyclonal antibody	MRC CVR	N/A
Anti-NSP16 sheep polyclonal antibody	MRC CVR	N/A
Anti-Actin antibody	Developmental Studies Hybridoma Bank JLA20	Cat. # 528068
PE-labeled anti-NGFR antibody	Biolegend	Cat. # 345105
Rabbit anti-SARS-Cov-2 Spike Antibody	Sino	Cat. # 40591-T62-100
PacBlue-labeled streptavidin	Thermo	Cat. # S1222
PE-labeled anti-Rabbit	Thermo	Cat. # P-2771MP
anti-puromycin antibody	EMD Millipore	Cat. # MABE343 Clone 12D10

**Bacterial and Virus Strains**		

2019-nCoV/USA_USA WA1/2020 (WA1)	WRCEVA	N/A

**Chemicals, Peptides, and Recombinant Proteins**		

HMW poly(I:C)	Invivogen	Cat. # tlrl-pic
IFN-beta	R&D Systems	Cat. # 8499-IF-010/CF
HaloTag® Alexa Fluor® 660 Ligand	Promega	Cat. # G8471
Halolink Resin	Promega	Cat. # G1915
Biotin Picolyl Azide	Click Chemistry Tools	Cat. # 1167-25
5 ethyl uridine	Jena Bioscience	Cat. # CLK-N002-CSTM

**Critical Commercial Assays**		

QUANTI-Blue	Invivogen	Cat. # rep-qbs
1-Step Human Coupled IVT-DNA	Thermo	Cat. # 88882
Rabbit Reticulocyte Lysate System	Promega	Cat. # L4960
TransIT-mRNA Transfection Kit	Mirus	Cat. # MIR2225
HiScribe T7 ARCA mRNA Kit	NEB	Cat. # E2060S

**Deposited Data**		

SARS-CoV-2 Protein Capture and RNA Sequencing	NCBI Short Read Archive (SRA)	Accession # PRJNA665692
Nascent and total RNA-Seq data	NCBI Short Read Archive (SRA)	Accession # PRJNA665581
Pre-catalytic human spliceosome structure	Charenton et al., 2019	PDB: 6QX9
Structure of S domain of 7SL	Kuglstatter et al., 2002	PDB: 1MFQ
Structure of SRP-Ribosome Complex	Voorhees and Hegde, 2015	PDB: 3JAJ
Structure of Ebp1-ribosome complex	Wild et al., 2020	PDB: 6SXO
Structure of the human 40S ribosome	Ameismeier et al., 2018	PDB: 6G5H
Structure of mRNA path through ribosome	Simonetti et al., 2020	PDB: 6YAL
Structure of SERBP1	Brown et al., 2018	PDB: 6MTE
Structure of Stm1	Ben-Shem et al., 2011	PDB: 4V88
APPRIS database	Rodriguez et al., 2013	N/A

**Experimental Models: Cell Lines**		

HEK293T	ATCC	Cat. # CRL-3216
Vero E6	J.L. Whitton and Michele Bouloy	N/A
HEK-Blue-ISG	Invivogen	Cat. # hkb-igs-1
A549	ATCC	Cat. # CCL-185
Calu3	ATCC	Cat. # HTB-55

**Oligonucleotides**		

Primers for In-vitro transcription	See Table S4	N/A
Primers used for qPCR	See Table S2	N/A
siRNA targeting SRP19	Dharmacon	L-019729-01-0005
siRNA targeting SRP54	Dharmacon	L-005122-01-0005

**Recombinant DNA**		

Template used in mRNA generation	See Table S5	N/A
NSP1	Kim et al., 2020a	RRID: Addgene_141255
NSP2	Kim et al., 2020a	RRID: Addgene_141256
PLPRO (NSP3)	Kim et al., 2020a	RRID: Addgene_141257
NSP4	Kim et al., 2020a	RRID: Addgene_141258
NSP5	Kim et al., 2020a	RRID: Addgene_141259
NSP6	Kim et al., 2020a	RRID: Addgene_141260
NSP7	Kim et al., 2020a	RRID: Addgene_141261
NSP8	Kim et al., 2020a	RRID: Addgene_141262
NSP9	Kim et al., 2020a	RRID: Addgene_141263
NSP10	Kim et al., 2020a	RRID: Addgene_141264
NSP11	Kim et al., 2020a	RRID: Addgene_151991
RNA-pol (NSP12)	Kim et al., 2020a	RRID: Addgene_141265
Heli (NSP13)	Kim et al., 2020a	RRID: Addgene_141266
NSP14	Kim et al., 2020a	RRID: Addgene_141267
NSP15	Kim et al., 2020a	RRID: Addgene_141268
NSP16	Kim et al., 2020a	RRID: Addgene_141269
ORF3A	Kim et al., 2020a	RRID: Addgene_141271
ORF3B	Kim et al., 2020a	RRID: Addgene_141272
E	Kim et al., 2020a	RRID: Addgene_141273
M	Kim et al., 2020a	RRID: Addgene_141274
ORF6	Kim et al., 2020a	RRID: Addgene_141275
ORF7A	Kim et al., 2020a	RRID: Addgene_141276
ORF7B	Kim et al., 2020a	RRID: Addgene_141277
ORF8	Kim et al., 2020a	RRID: Addgene_141278
ORF9B	Kim et al., 2020a	RRID: Addgene_141280
N	Kim et al., 2020a	RRID: Addgene_149330
NSP11 (pGBW-m4133457)	Ginko Bioworks	RRID: Addgene_151991
pB-TAG-ERN	Kim et al., 2016	RRID: Addgene_80476
NGFR	Izon et al., 2001	RRID: Addgene_27489

**Software and Algorithms**		

Imaris	Oxford Instruments, Imaris	https://imaris.oxinst.com
Transform-restrained Rosetta Algorithm	Yang et al., 2020	https://yanglab.nankai.edu.cn/trRosetta/
Pymol	Schrodinger	https://pymol.org/2/
Modeler version 9.24	Webb and Sali, 2016	https://salilab.org/modeller/manual/
FloJo analysis software	FlowJo, LLC.	https://www.flowjo.com/solutions/flowjo
MAFFTT (v7.407)	Rozewicki et al., 2019	https://mafft.cbrc.jp/alignment/server/
Geneious	Geneious	https://www.geneious.com
Integrative Genomics Viewer	Robinson et al., 2011	http://software.broadinstitute.org/software/igv/
Morpheus	Broad Institute	https://software.broadinstitute.org/morpheus/
Molecular Signatures Database Gene Ontology Biological Processes and Reactome	Liberzon et al., 2015	https://www.gsea-msigdb.org/gsea/msigdb
Trimmomatic	Bolger et al., 2014	https://github.com/timflutre/trimmomatic
STAR Aligner	Dobin et al., 2013	https://github.com/alexdobin/STAR
PICARD	“Picard Toolkit.” 2019. Broad Institute, GitHub Repository.	https://broadinstitute.github.io/picard/
Bowtie2	Langmead and Salzberg, 2012	http://bowtie-bio.sourceforge.net/bowtie2/index.shtml

### Resource Availability

#### Lead Contact

Further information and requests for reagents and resources should be directed to and will be fulfilled by the Lead Contact, Mitchell Guttman (mguttman@caltech.edu).

#### Materials Availability

All constructs and plasmids generated in this study will be made available on request sent to the Lead Contact with a completed Materials Transfer Agreement.

#### Data and Code Availability

All datasets generated during this study are available at NCBI Short Read Archive: Bioproject PRJNA665692 (viral protein purifications) and PRJNA665581 (nascent and total RNA-Seq). Table S1 is available at Mendeley data archive, Mendeley Data: http://dx.doi.org/10.17632/zg7wp4xd5v.1

### Experimental Model and Subject Details

#### Cell lines used in this study

We used the following cell lines in this study: (i) HEK293T, a female human embryonic kidney cell line obtained from ATCC. (ii) HEK-Blue ISG, Interferon regulatory factor (IRF)-inducible Secreted Alkaline Phosphatase (SEAP) reporter HEK293 cells of female origin (Invivogen). (iii) A549, a male human lung epithelial cell line obtained from ATCC. (iii) Calu3, a male human lung epithelial cell line obtained from ATCC, (iv) Vero E6, a female African green monkey kidney cell line, kindly provided by J.L. Whitton and Michele Bouloy.

#### Cell culture conditions

A549s, HEK293T cells and derivatives were cultured in complete media consisting of DMEM (GIBCO, Thermo Fisher Scientific) supplemented with 10% FBS (Seradigm Premium Grade HI FBS, VWR), 1X penicillin-streptomycin (GIBCO, Thermo Fisher Scientific), 1X MEM non-essential amino acids (GIBCO, Thermo Fisher Scientific), 1 mM sodium pyruvate (GIBCO, Thermo Fisher Scientific) and maintained at 37°C under 5% CO_2_. For maintenance, 800,000 cells were seeded into 10 mL of complete media every 3-4 days in 10 cm dishes. Vero E6 cells were maintained in complete DMEM (Thermo Fisher Scientific, 11965–092) containing 10% fetal bovine serum (FBS) (Thermo Fisher Scientific, 16140–071), 1% HEPES Buffer Solution (Thermo Fisher Scientific, 15630–130), and 1% penicillin-streptomycin (Thermo Fisher Scientific, 15140–122). Calu3 cells were maintained in Eagles’s Minimal Essential Medium (ATCC) containing 10% FBS and 1% penicillin-streptomycin purchased from Thermo Fisher Scientific. All cell lines were maintained at 37°C under 5% CO_2_. Cells were grown in a humidified incubator at 37°C with 5% CO_2_.

#### SARS-CoV-2 Viral Infection strains and conditions

All experiments using infectious SARS-CoV-2 conducted at the UVM BSL-3 facility were performed under an approved Institutional Biosafety protocol. SARS-CoV-2 strain 2019-nCoV/USA_USA WA1/2020 (WA1) was generously provided by Kenneth Plante and the World Reference Center for Emerging Viruses and Arboviruses (WRCEVA) at the University of Texas Medical Branch and propagated in Vero E6 cells. Viral infections were performed at the indicated multiplicity of infection in a low volume of normal cellular maintenance media containing 2% FBS for one hour at 37°C, inoculum was removed and then overlaid in the respective cellular maintenance media containing 10% FBS for the indicated time periods. Experiments performed to visualize the location of viral NSP proteins (and associated antibody validation) were performed in a Containment Level 3 facility at the MRC-University of Glasgow Centre for Virus Research using SARS-CoV-2 strain England-02 (from Public Health England [now called National Institute for Health Protection], GISAID: EPI_ISL_407073) using a MOI of 0.1 or 1 (as indicated).

### Method Details

#### Generation of RNA binding maps

##### Cloning of expression constructs

SARS-CoV-2 protein constructs (with the exception of Nsp11) were a gift from Fritz Roth (see Table S3 for Addgene information) (Kim et al., 2020a) and were LR-cloned (Invitrogen Gateway Cloning, Thermo Fisher Scientific) into mammalian expression destination vector pCAG-Halo-TEV-DEST-V5-IRES-puroR. Note that following LR cloning, proteins were not V5-tagged because all entry clones contained stop codons. For NSP11, an entry clone was generated by BP cloning (Invitrogen Gateway Cloning, Thermo Fisher Scientific) a PCR amplicon (primers: ggGGACAAGTTTGTACAAAAAAGCAGGCTTTtcagctgatgcacaatcgtttttaaacgg and gGGGACCACTTTGTACAAGAAAGCTGGGTTttacaccgcaaacccgtttaaaaacgattg; template: pGBW-m4133457 a gift from Ginkgo Bioworks) into pDONR221.

##### Expression and lysis

For each viral protein capture, we transfected 10 μg of these expression vectors into HEK293T cells grown on a 15cm dish using BioT transfection reagent (Bioland) according to manufacturer’s recommendations. 24-48 hours post-transfection, cells were washed once with PBS and then crosslinked on ice using 0.25 J cm^−2^ (UV2.5k) of UV at 254 nm in a Spectrolinker UV Crosslinker. Cells were then scraped from culture dishes, washed once with PBS, pelleted by centrifugation at 1,000 *× g* for 5 min, and flash-frozen in liquid nitrogen for storage at –80°C. We lysed batches of 5 million cells by completely resuspending frozen cell pellets in 1 mL of ice cold lysis buffer (50 mM HEPES, pH 7.4, 100 mM NaCl, 1% NP-40, 0.1% SDS, 0.5% Sodium Deoxycholate) supplemented with 1X Protease Inhibitor Cocktail (Promega), 200 U of Ribolock (Thermo Fisher Scientific), 20 U Turbo DNase (Ambion), and 1X Manganese/Calcium Mix (0.5mM CaCl_2_, 2.5 mM MnCl_2_). Samples were incubated on ice for 10 minutes to allow lysis to proceed. The lysates were then incubated at 37°C for 10 minutes at 700 rpm shaking on a Thermomixer (Eppendorf). Lysates were cleared by centrifugation at 15,000 *× g* for 2 minutes. The supernatant was collected and kept on ice until bound to the HaloLink Resin (Promega). Of the 1mL lysis volume, 50uL was set aside for input, 20uL used for protein expression confirmation, and the rest for capture on HaloLink Resin as described below.

##### Expression confirmation

Lysis supernatant set aside for expression testing was combined with 1.5 μL of 1:60 diluted HaloTag® Alexa Fluor® 660 Ligand (Promega) and incubated at room temperature for 20 min in the dark. To stop the reaction, we added LDS loading buffer to 1X final concentration (4 μL 10X Bolt reducing agent, 10 μL 4X NuPAGE® LDS Sample Buffer, 4 μL H_2_O), denatured the mixture at 90°C for 10 min and ran on a Bolt 4%–12% Bis-Tris Plus Gel (all products Thermo Fisher Scientific). Resolved gel was imaged directly on a Li-Cor Odyssey CLx.

##### Protein capture

We used 200 μL of 25% HaloLink Resin slurry (50 μL of HaloLink Resin total) per 5 million cells. Resin was washed three times with 2 mL of 1X PBS-T (1x PBS + 0.1% Triton X-100) and incubated in 1X Blocking Buffer (50 mM HEPES, pH 7.4, 100 μg/mL BSA) for 20 minutes at room temperature with continuous rotation. After the incubation, resin was washed three times with 1X PBS-T. The cleared lysate was mixed with 50 μL of HaloLink Resin and incubated at 4°C for 3-16 hr with continuous rotation. The captured protein bound to resin was washed three times with lysis buffer at room temperature and then washed three times at 90°C for 3 minutes while shaking on a Thermomixer at 1200 rpm with each of the following buffers: 1X NLS buffer (1xPBS, 2% NLS, 10 mM EDTA), High Salt Buffer (50 mM HEPES, pH 7.4, 0.1% NP-40, 1M NaCl), 8M Urea Buffer (50 mM HEPES, pH 7.5, 0.1% NP-40, 8 M Urea), Tween buffer (50 mM HEPES, pH 7.4, 0.1% Tween 20) and TEV buffer (50 mM HEPES, pH 7.4, 1 mM EDTA, 0.1% NP-40). The extended incubation of the bound RNA with the wash buffers leads to chemical fragmentation of the RNA yielding sizes that are suitable for RNA library preparation and binding site resolution. Between each wash, samples were centrifuged at 1,000 *× g* for 30 s and supernatant was removed. After the last wash, samples were centrifuged at 7,500 *× g* for 30 s and supernatant was discarded. For elution, the resin was resuspended in 100 μL of NLS Buffer and 10 μL of Proteinase K (NEB) and the sample was incubated at 50°C for 30 minutes while shaking at 1200 rpm. Input samples were similarly digested. Capture reactions were transferred to microspin cups (Pierce, Thermo Fisher Scientific), centrifuged at 2,000 *× g* for 30 s, and elutions used for RNA purification by RNA Clean and Concentrate-5 kits (Zymo, > 17nt protocol).

For qPCR analysis, cDNA was generated from purified RNA using Maxima H- reverse transcriptase (Thermo Fisher Scientific) following manufacturer’s recommendations. Amplification reactions were assembled with primer sets indicated in Table S2 and LightCycler® 480 SYBR Green I Master (Roche) following manufacturer’s protocols and read out in a Roche Lightcycler 480.

##### Library construction

RNA-Seq libraries were constructed from purified RNA as previously described (Van Nostrand et al., 2016). Briefly, after proteinase K elution, the RNA was dephosphorylated (Fast AP) and cyclic phosphates removed (T4 PNK) and then cleaned using Silane beads as previously described (Van Nostrand et al., 2016). An RNA adaptor containing a RT primer binding site was ligated to the 3′ end of the cleaned and end-repaired RNA. The ligated RNA was reverse transcribed (RT) into cDNA, the RNA was degraded using NaOH, and a second adaptor was ligated to the single stranded cDNA. Library preparation was the same for input samples except that an initial chemical fragmentation step (90°C for 2 min 30 s in 1X FastAP buffer) was included prior to FastAP treatment. This chemical fragmentation step was designed to be similar to the fragmentation conditions used for purified Halo bound samples. The DNA was amplified and Illumina sequencing adaptors were added by PCR using primers that are complementary to the 3′ and 5′ adapters. The molarity of PCR amplified libraries were measured by Agilent Tapestation High Sensitivity DNA screentapes and all samples were pooled at equal molarity. The pool was then purified and size selected on a 2% agarose gel and cut between 150-700 nts. The final libraries were measured by Agilent Bioanalyzer and Qubit high sensitivity DNA to determine the loading density of the final pooled sample. Pooled samples were paired-end sequenced on an Illumina HiSeq 2500 with read length 35 × 35nts.

#### Antibody Generation

To generate the sheep polyclonal anti-NSP1, anti-NSP8, anti-NSP9, and anti-NSP16 antibodies utilized in this study, NSP1, NSP8, NSP9 and NSP16 (using QHD43415.1 as reference) were cloned into pGex (GST-tagged) and pMex (MBP-tagged), in order to produce GST- and MBP-tagged respective NSP proteins. The N-terminal GST fusions were then used as antigens to immunize sheep. A bleed from the sheep was taken 7 days later, after which the MBP-tagged NSP proteins were used for serum affinity purification of the antibodies. To validate expression of the antibodies, Vero E6 cells were uninfected (mock) or infected with SARS-CoV-2 England-02 using a MOI of 0.1 or 1 (as indicated). At 72 hours post infection, the samples were harvested and the resulting whole cell lysates were probed by western blot with either sheep anti-NSP or mouse anti-actin (Developmental Studies Hybridoma Bank JLA20, antibody registry ID: AB_528068) primary antibodies.

#### Microscopy imaging

Cells were seeded on gelatin/laminin and poly-D-lysine (Sigma) coated coverslips or chamber slides (Nunc, Thermo Fisher Scientific) and transfected with mammalian expression vectors for Halo-tagged viral proteins. After 16-24 hours, cells were incubated with TMR-HaloTag® Ligand (Promega) according to manufacturer’s instructions, washed with PBS and fixed in 4% Formaldehyde (Pierce, Thermo Fisher Scientific). Cells were subsequently incubated in DAPI for 10 min and washed with PBS. For chamber slides, samples were imaged directly. For coverslips, samples were washed with water and mounted with ProLong Gold + DAPI (Molecular Probes, Thermo Fisher Scientific). We acquired images on a Nikon TS100-F widefield microscope or a Zeiss LSM800 inverted confocal microscope, collecting in line-scanning mode with 4x line averaging using a 63x oil objective.

For staining of infected cells, cells were fixed and permeabilized in 8% formaldehyde 1% Triton, and subsequently labeled with primary antibodies raised in sheep to SARS-CoV-2 at 1/500 dilution, followed by incubation with a rabbit anti-sheep Alexa 555 secondary antibody (Abcam, ab150182) at 1/1000 dilution and mounted with DAPI in the medium (Thermo Fisher Scientific, cat# P36395). Cells were imaged with a Zeiss LSM 880 confocal microscope, with 1 Airy unit pinhole for all primary antibody channel acquisitions and pixel size 0.07 μm x 0.07 μm. The objective lens used was a Zeiss Plan-Apochromatic 63x/1.4NA M27.

#### Structure modeling

##### NSP1 homology model

The predicted model of SARS-CoV-2 NSP1 was generated using the transform-restrained Rosetta (trRosetta) algorithm, a deep learning-based modeling method based on the Rosetta energy minimization pipeline with additional distance and interaction restraints generated from co-evolution (Yang et al., 2020). All figures were generated using Pymol (https://pymol.org/2/).

##### NSP1-ribosome model

The model of NSP1 bound to the ribosome was generated using Modeler version 9.24(Webb and Sali, 2016). The C-terminal sequence of NSP1 (KHSSGVTRELMRELNGG) was modeled using the structure of SERBP1 bound to the ribosome (PDB ID: 6MTE, chain w) as a template. The default Modeler parameters were used to create an alignment of NSP1 and SERBP1 and to generate the model, and all atoms within 6Å of SERBP1 were included in the model to define the neighboring environment. Twenty models were generated and the model with the lowest DOPE score was selected to visualize with Pymol (Delano, 2002).

##### Structural analysis of protein-RNA interactions

X-ray crystal structures and cryo-electron microscopy structures were obtained from the Protein Data Bank (http://www.rcsb.org; Berman et al., 2000) and visualized with PyMOL (Delano, 2002). For U1 and U2 structural analysis, we used a cryo-EM structure of the pre-catalytic human spliceosome (PDB ID: 6QX9). For 7SL structural analysis, we used an X-ray crystal structure of the human signal recognition particle (PDB ID: 1MFQ). To examine human SRP in the context of the ribosome, we used a cryo-EM structure of the mammalian SRP-ribosome complex (PDB ID: 3JAJ). To analyze the ribosomal ES27 expansion segment, we superimposed a cryo-EM structure of the expansion segment (PDB ID: 6SXO) onto the complete ribosome structure (PDB ID: 3JAJ) using the PyMOL command “super.” Finally, for NSP1–18S rRNA structural analysis, we used multiple structures of the ribosome, including structures of the pre-40S subunit (PDB ID: 6G5H), 48S late-stage initiation complex (PDB ID: 6YAL), 80S in complex with SERBP1 (PDB ID: 6MTE), and 80S in complex with Stm1 (PDB ID: 4V88).

#### Recombinant NSP1 production

NSP1 was cloned into a bacterial expression vector resulting in N-terminally tagged Halo-6xHis-tagged Nsp1. The NSP1 sequence was PCR amplified from Addgene Nsp1 entry vector to add a N-terminal 6X HIS tag and restriction enzyme sites for digestion and ligation into N-terminal Halo bacterial expression vector. This construct was transformed into BL21 DE3 *E. coli* (Agilent), expanded to a 500mL liquid culture, and grown until OD_600_ reached 1.0. IPTG was added to a final concentration of 1mM. After 3 hours of IPTG induction, bacteria was centrifuged for 15 min at 5000 *× g*. Pellet was lysed with binding buffer (50mM HEPES, pH 7.5, 20mM MgCl_2_, 600mM NaCl, 2mM TCEP, 10mM Imidazole, 2mM ATP, 1% Triton X-100) supplemented with ATP (2mM), protease inhibitor cocktail (Promega), Benzonase (Sigma) and Triton X-100 (Sigma) using 5mL of lysis mix per gram of wet cell paste. Cell suspension was rocked for 20 min at room temperature and then centrifuged at 16,000 *× g* for 20 min at 4°C. Supernatant was incubated with washed iMAC resin (Bio-Rad) and rocked for 20 min at room temperature. We loaded the resin-lysate mixture into an appropriately-sized column and washed with 5 column volumes of binding buffer (50mM HEPES, pH 7.5, 20mM MgCl_2_, 600mM NaCl, 2mM TCEP, 10mM Imidazole, 2mM ATP, 1% Triton X-100) followed by 10 column volumes of wash buffer (50mM HEPES, pH 7.5, 600mM NaCl, 2mM TCEP, 20mM Imidazole, pH 8). Recombinant NSP1 (rNSP1) was eluted with 5 column volumes of elution buffer by adding 1 column volume at a time with column flow stopped, collecting eluate after each addition, and waiting 15 min between each elution buffer addition. We dialyzed these eluates with a 10mL Spectra-Por® Float-A-Lyzer® G2 (Spectrum Laboratories) into storage buffer (50mM HEPES, pH 7.5, 150mM NaCl, 10% glycerol) at 4°C using 2 exchanges, one after 2 hours and then overnight.

#### *In vitro* translation assays

Pierce 1-Step Human Coupled IVT-DNA (Thermo Fisher Scientific) *in vitro* translation kit was used to measure rNsp1-dependent translation inhibition. Bovine Serum Albumin (BSA), and buffer only controls were used to control for the addition of excess protein or changes in buffer composition. To measure translation inhibition, 5 μL *in vitro* translation reactions were assembled, scaled according to manufacturer’s recommendations. The included control plasmid pCFE-GFP was used to measure translational output of the reactions. GFP fluorescence was measured on a BioTek Cytation3 plate reader using emission filters for GFP fluorescence. 1.5 μM stock dilutions of rNsp1 and BSA were made in storage buffer (50mM HEPES, pH 7.5.,150mM NaCl,10% glycerol). Subsequent 10-fold dilutions were made in storage buffer to span a concentration range of 1000 nM to 1 nM for each protein in the final reaction. 10 μL of the diluted protein solution was added to the 5 μL translation reactions, and incubated for 5 minutes at room temperature prior to the addition of the GFP reporter plasmid. Duplicate reactions were made to measure variability for each condition. In addition, a buffer only control was included to measure the effect of dilution of the translation reaction by the storage buffer. After the 5 minute incubation, 50 ng of GFP reporter plasmid was added to each reaction and incubated at 30°C for 4 hours prior to fluorescence detection. Two microliters from each reaction was measured in duplicate on a Biotek Cytation3 microplate reader using excitation and emission filters for GFP. Sample readings were blanked by subtracting values obtained from the buffer only control.

Promega’s Rabbit Reticulocyte Lysate System was also used to assay translation inhibition. To measure translation inhibition, 10 μL *in vitro* translation reactions were assembled, scaled according to manufacturer’s recommendations. For each translation reaction, either 10 μL of recombinant protein storage buffer or rNSP1 was added, followed by 500ng of mRNA. After 4 hours of incubation at 30°C, luciferase was read out using the Bright-Glo luciferase assay (Promega) or GFP fluorescence was measured, both on a Biotek Cytation3 plate reader.

#### *In vivo* translation assays

We assayed translation in HEK293T cells transfected with mammalian expression vectors, mRNAs, or combinations of these. For mRNA transfections of fluorescence protein translation reporters (including unmodified, +SARS-CoV2 leader sequence, +SL1, +SL2-SL1, and +5nts), DNA templates for *in vitro* transcription were generated with sequences appended to the 5′ end of GFP and mCherry (see Tables S4 and S5 for primers and templates, respectively) and transcribed using HiScribe T7 ARCA mRNA Kit with tailing (New England Biolabs). For Nsp1 mRNA transfection, indicated primers from Table S4 were used to add restriction enzyme sites for cloning into pT7CFE1-CHis backbone provided in the Pierce Human 1-step Coupled IVT Kit and HiScribe T7 High Yield RNA Synthesis Kit (New England Biolabs) was used for *in vitro* transcription.

Using BioT transfection reagent, mammalian expression vectors for a GFP reporter and for SARS-CoV-2 viral proteins were transfected into HEK293T cells seeded for imaging, as described above, or seeded in 24 well plate format. To transfect only mRNA, Lipofectamine messengerMax (Invitrogen, Thermo Fisher Scientific) or *Trans*IT-mRNA Transfection Kit (Mirus Bio) was used. For transfections that included both mRNA and plasmid, Lipofectamine 2000 (Invitrogen, Thermo Fisher Scientific) was used.

To measure fluorescence at 24 (leader-mCherry, no leader-GFP) or 48 hours (leader GFP, no-leader mCherry) post-transfection, cells were trypsinized and processed for flow cytometry or transferred into black 96 well plates (Nunc) for fluorescence detection on a Biotek Cytation 3 plate reader. For flow cytometry, lifted cells were washed with CBH buffer (10mM HEPES, pH 7.4, 0.5% BSA, Hank’s Balanced Salt Solution (GIBCO, Thermo Fisher Scientific)), resuspended with a viability dye (7-AAD or DAPI) and analyzed on a MACSQuant Vyb. Acquistion files were analyzed with FlowJo analysis software.

#### SUnSET assay

To assay global protein translation, a SUnSET assay was performed as previously described (Schmidt et al., 2009). Mammalian expression vectors were exchanged for versions that did not confer puromycin resistance and thus, for these experiments, LR reactions were carried out with destination vector pB-Halo-DEST-IRES-NGFR. Resulting expression vectors drive protein expression by a dox-inducible promoter, contain the rtTA needed for dox induction, and produce an N-terminally-tagged Halo fusion protein. Generation of this destination vector made use of the pB-TAG-ERN backbone (a gift from Knut Woltjen; Addgene plasmid # 80476; http://addgene.org/80476; RRID:Addgene_80476)(Kim et al., 2016) and the NGFR (Truncated Human Nerve Growth Factor Receptor) coding sequence from Addgene plasmid #27489 (a gift from Warren Pear; http://addgene.org/27489; RRID:Addgene_27489)(Izon et al., 2001).

We transfected these mammalian expression vectors for NSP1 and GFP into HEK293T using BioT transfection reagent. After 3 hours, doxycycline (Sigma) was added to a final concentration of 2 μg/mL. After 24 hours, cells were incubated with puromycin (10 μg/mL) for 10 min, then washed with fresh media, and harvested with cold PBS. Pelleted cells were lysed for 10 min on ice (mixing after 5 min) with 100uL RIPA buffer supplemented with protease inhibitor cocktail (Promega). Insoluble debris was pelleted by centrifuging at 12,500 *× g* for 2.5 minutes and supernatant was run on a Bolt 4%–12% Bis-Tris Plus Gel (Thermo Fisher Scientific). Proteins were then transferred to nitrocellulose using the iBlot transfer system (Thermo Fisher Scientific) and western blotting carried out using an anti-puro antibody (clone 12D10, EMD Millipore).

#### SUnSET in SARS-CoV-2 infected cells

SUnSET in SARS-CoV-2 infection was performed as above with the following modifications. Cells were infected or not (mock) with SARS-CoV-2, and 48 hpi cells were incubated with puromycin (10 μg/mL) for 20 min. Media was aspirated and cells lysed directly in 2X Laemmli’s buffer (Biorad), heated at 95°C for ten minutes and run on a 4%–12% NuPAGE Gel (Thermo Fisher Scientific). Proteins were transferred to nitrocellulose using the iBlot transfer system and probed as above.

#### Membrane protein reporter experiments

To assay SRP-dependent membrane protein transport to the cell surface, we monitored surface arrival of exogenously expressed Neuronal Growth Factor Receptor (NGFR) by flow cytometry in the presence of NSPs. Mammalian expression vectors were exchanged for versions that contained an IRES-NGFR to co-express a membrane reporter and thus, for these experiments, LR reactions were carried out with destination vector pB-6xHis-GFP-DEST-IRES-NGFR. Resulting expression vectors drive protein expression by a dox-inducible promoter, contain the rtTA needed for dox induction, and produce an N-terminally-tagged His-GFP fusion protein and a co-expressed NGFR. The GFP here is an enhanced GFP containing an amino acid substitution (A205K) to generate a monomeric variant based on previous literature (Alberti et al., 2018).

We transfected these mammalian expression vectors for NSP8, NSP9, NSP1ΔRC mutant and EED into HEK293T using BioT transfection reagent, induced expression with 2 μg/ml doxycycline 24 hours after transfection, and assessed surface arrival of NGFR 24 hours after induction. To carry out flow cytometric analysis, cells were lifted with 1mM EDTA, washed once with PBS and stained with PE-labeled anti-NGFR antibody (Biolegend; 1/600 dilution in PBS, 0.5%BSA) and analyzed on a MACSQuant Vyb. Fluorescence intensity measurements were taken for GFP and PE and analyzed using FloJo analysis software.

#### siRNA experiments for SRP19 and SRP54

To knockdown SRP19 and SRP54, siRNAs targeting each (Dharmacon cat# L-019729-01-0005 and L-005122-01-0005, respectively) were transfected into HEK293T cells using Lipofectamine RNAiMAX (Invitrogen) according to manufacturer’s protocols. To validate knockdown, transfected cells were assayed by qPCR using primer sets (Table S2) to amplify each target as well as normalizer Calm3. Transfections were carried out 48 hours prior to assaying cells, either by qPCR, membrane reporter, or membrane SUnSET (see below) experiments.

#### Leader-NGFR measurements

Calu3 and Vero cells were transfected with mRNAs encoding leader-NGFR and leader-GFP using TransIT-mRNA Transfection Kit (Mirus) and subsequently infected with SARS-CoV-2 at an MOI of 0.1. After 24 hours, cells were washed with PBS, trypsinized and fixed in 4% PFA for 20 minutes before staining with biotinylated anti-NGFR (BioLegend) and anti-SARS-Cov-2 Spike Antibody (Sino) and subsequently stained with PE-labeled anti-Rabbit (Thermo, P-2771MP) and PacBlue-labeled streptavidin (Thermo, S1222). FACS was performed on a MACSquant Flow cytometer and analyzed using FloJo analysis software; FACS distributions were compared using a 2-tailed Kolmogorov-Smirnov test. For these experiments, RNA was transcribed from a PCR template (see Table S4) using the HiScribe T7 ARCA mRNA kit (with tailing).

#### Membrane SUnSET assay

To assay transport to the cell surface of all plasma membrane proteins, the SUnSET assay was adapted to puro-label surface proteins as previously described (Schmidt et al., 2009), and read out by flow cytometry. Briefly, cells were incubated with puromycin as described above, followed by two quick washes and a chase with fresh complete media for 50 min. Cells were lifted with 1mM EDTA as described above and stained with an anti-puro antibody (clone 12D10, EMD Millipore) conjugated to Alexa-647. For these experiments, NSP was expressed from the same vector described above for membrane reporter assays. Fluorescence intensity measurements were taken for GFP and Alexa-647 on a MACSquant Flow cytometer and analyzed using FloJo analysis software; distributions were compared using a 2-tailed Kolmogov-Smirnov.

#### Splicing assessment experiments

##### IRF7-GFP splicing reporter

To assess splicing efficiency, exons 5-6 of mouse IRF7 (ENMUST00000026571.10) containing its endogenous intron were fused upstream of 2A self-cleaving peptide and eGFP and cloned into an MSCV vector (PIG, Addgene) (Mayr and Bartel, 2009). This construct was co-transfected into HEK293Ts with NSP16 or GFP and measured 24 hours after transfection by flow cytometry (Macsquant) and analyzed using FloJo analysis software.

##### 5EU labeling of RNA

SARS-Cov2 or mock infected Calu3 cells and Nsp16- or GAPDH-expressing HEK293Ts were labeled with 5-Ethynyl-uridine (5EU; Jena Bioscience) by adding 5EU containing media to cells for 20 min at a final concentration of 1mM, as previously described (Jao and Salic, 2008). After the pulse label, cells were washed with warm PBS and lysed in RLT buffer (QIAGEN). Total RNA was isolated from cells using manufacturer’s protocols for Qiashredder and RNeasy RNA isolation (both QIAGEN), followed by Turbo DNase treatment (Ambion, Thermo Scientific), and Zymo RNA Clean and Concentrate. For each sample, 2 μg of RNA was used for ligation of a unique barcoded RNA adaptor, following the relevant steps in the protocol described above in Library Construction of RNA-seq libraries. Samples were then pooled before proceeding to biotinylation steps.

##### Biotinylation of 5EU labeled RNA

To biotinylate 5EU-labeled RNA, samples were first mixed, in order, with water, HEPES (100 mM), biotin picolyl azide (1 mM; Click Chemistry Tools) and Ribolock RNase inhibitor, then added to premixed CuSO_4_ (2 mM) and THPTA (10mM), and finally added to freshly prepared sodium ascorbate (12mM), as previously described (Hong et al., 2009). The click reaction was incubated for 1 hour at 25°C with 1000rpm shaking on an Eppendorf thermomixer followed by RNA purification using > 17nt protocol for Zymo Clean and Concentrate.

##### Sequential capture of biotinylated RNA

We completed three rounds of sequential capture on streptavidin beads to isolate nascent transcripts (see Figure S3B). To capture biotinylated RNA, MyOne Streptavidin C1 Dynabeads (ThermoFisher Scientific) were first washed three times in Urea buffer (10mM HEPES, pH 7.5, 10mM EDTA, 0.5M LiCl, 0.5% Triton X-100, 0.2% SDS, 0.1% sodium deoxycholate, 2.5mM TCEP, 4M Urea) followed by three additional washes in M2 buffer (20mM Tris, pH 7.5, 50mM NaCl, 0.2% Triton X-100, 0.2% sodium deoxycholate, 0.2% NP-40). Washed beads were mixed with 3 parts 4M Urea buffer and 1 part biotinylated RNA and incubated for 60 min with 900rpm thermomixer shaking at room temperature.

After magnetic separation, beads were washed 3 times with M2 buffer followed by 3 washes with Urea buffer at 37°C at 750rpm for 5 min. RNA was eluted from beads in 2 sequential elutions by incubating with elution buffer (5.7M guanidine thiocyanate, 1% N-lauroylsarcosine; both Sigma) at 65°C for 2 minutes, repeating with more elution buffer for a second elution.

The elutions were pooled, diluted with Urea buffer, incubated with pre-washed streptavidin beads, washed, and eluted for 2 additional rounds exactly as described above for a total of 3 sequential captures.

Final elutions were pooled, cleaned with Zymo RNA Clean and Concentrate following manufacturer’s protocols, and carried through RNA-seq library preparation as described above starting with the reverse transcription step.

#### Interferon stimulation experiments

HEK-Blue ISG cells were seeded in 96 well plates, transfected with Nsp1 mammalian expression vectors using BioT and stimulated with 50 ng/ml human IFN-B (R&D Systems). Supernatants were assayed for alkaline phosphatase as per manufacturer instructions using QUANTI-Blue reagent (Invivogen).

HEK293T cells were seeded in 6 well plates, transfected with either Halo-tagged GapdH, Nsp1, NSP8 and NSP9 in combination, or NSP16 mammalian expression vectors using BioT. 24 hours later, the media was replaced with media containing 50 ng/ml human IFN-β (R&D Systems). Expression was assayed using live cell Halo-imaging. Halo-TMR ligand was diluted 1:200 in media and added to the culture for a 1:1000 final dilution. Samples were incubated 30 minutes at 37°C, 5% CO_2_ and then the media was aspirated. Wells were rinsed twice with PBS, then media was added back to the wells. Samples were incubated 30 minutes at 37°C, 5% CO_2_ to allow uncoupled ligand to diffuse out of the cells. Media was then aspirated and replaced, and cells were imaged by widefield fluorescence microscopy. Cultures were ultimately harvested for RNA 24 hours later, or 48 hours post transfection.

A549s were seeded in 6 well plates, transfected with NSP1 mammalian expression vectors using Lipofectamine 2000 and stimulated with 1 μg/ml HMW poly(I:C) (Invivogen) 24h after transfection. Supernatant was assayed for secreted IFN-β by ELISA (Human IFN Beta ELISA, High Sensitivity, PBL) 24 hours after stimulation, and RNA from cells was purified and assessed for ISG gene expression as normalized to GAPDH expression (SYBR Green Master Mix, Bio-Rad). Primers used for qPCR are listed in Table S2.

#### 5′ viral leader experiments

Sars-CoV-2 Leader sequence was appended to the 5′ end of GFP and mCherry reporter templates via PCR. PCR templates were then transcribed using HiScribe T7 ARCA mRNA kit (with tailing). Leader mutants, including SL1 only, SL1/SL2 swap, and +5nts mutants were likewise appended to the 5′ end of fluorescent reporter templates via PCR and transcribed using Hiscribe T7 ARCA kit. mRNA reporters were transfected in HEK293T cells with Lipofectamine MessengerMax. To measure fluorescence of mCherry and GFP reporters, 24 hours post transfection cells were either lifted with PBS and transferred into black 96 well plates for fluorescence readout on a Biotek Cytation 3 or trypsinized and processed for flow cytometry.

#### Alignments and phylogeny reconstructions

Alignments were performed with MAFFTT (v7.407) using a local alignment (linsi–ep 0.123–reorder [in.fasta] > [out.aln.fasta]). Resulting alignments were visualized with Geneious. Pairwise distance matrices were visualized with Morpheus. Phylogeny reconstructions were performed with IQTREE multicore (v1.6.12), model selection with 1000 bootstrap pseudoreplicates (iqtree -s [out.aln.fasta] -m TEST -bb 1000 -nt 4 -o [outgroup]). Phylogenies were visualized with FigTree.

### Quantification and Statistical Analysis

#### Analysis of SARS-CoV-2 viral protein binding to RNA

##### Sequence alignment and analysis

For Halo purifications and RNA binding mapping sequencing reads were aligned to a combined genome reference containing the sequences of structural RNAs (ribosomal RNAs, snRNAs, snoRNAs, 45S pre-rRNA) and annotated mRNAs (RefSeq hg38) using Bowtie2. To distinguish between the nascent pre-ribosomal RNA and mature 18S, 28S, and 5.8S rRNA, we separated each of the components of the 45S into separate sequence units for alignment (e.g., ITS, ETS). We excluded all low quality alignments (MAPQ < 2) from the analysis. For mRNA analysis, we removed PCR duplicates using the Picard MarkDuplicates function (https://broadinstitute.github.io/picard/).

For each RNA, we enumerated 100 nucleotide windows across the entire RNA. For each window, we calculated the enrichment by computing the number of reads overlapping the window in the protein elution sample divided by the total number of reads within the protein elution sample. We normalized this ratio by the number of reads in the input sample divided by the total number of reads in the input sample. Because all windows overlapping a gene should have the same expression level in the input sample (which represents RNA expression), we estimated the number of reads in the input as the maximum of either (i) the number of reads over the window or (ii) the median read count over all windows within the gene. This approach provides a conservative estimation of enrichment because it prevents windows from being scored as enriched if the input values over a given window are artificially low, while at the same time accounting for any non-random issues that lead to increases in read counts over a given window (e.g., fragmentation biases or alignment artifacts leading to non-random assignment or pileups).

We calculated a multiple testing corrected *p-value* using a scan statistic, as previously described (Guttman et al., 2009, 2010). Briefly, *n* was defined as the number of reads in the protein elution plus the number of reads in the control sample. *p* was defined as the total number of reads in the protein elution sample divided by the sum of the protein elution sample total reads and total reads in the control sample. *w* was the size of the window used for the analysis (100 nucleotides). The scan statistic *p-value* was defined using the Poisson estimations based on standard distributions previously described (Naus, 1982).

Because RNA within input samples are fragmented differently than the protein elution samples, we noticed that the overall positional distribution of protein elution samples was distinct from Input distributions. Accordingly, we used the remaining protein elution samples (rather than Input) as controls for each protein. Specifically, this enabled us to test whether a given protein is enriched within a given window relative to all other viral and control proteins. Enrichments were computed as described above. These values are plotted in Figure 1 and Table S1.

##### Plotting and visualization

Enrichment plots for specific RNAs were visualized in IGV (Robinson et al., 2011) and were generated by either: (i) computing the enrichment for each nucleotide as described above. In this case, the read count for each nucleotide was computed as the total number of reads that overlapped the nucleotide. (ii) Counting the number of RT stop sites at a given nucleotide. In this case, we compute the alignment start position of the second in pair read and computed a count of each nucleotide. We normalized this count by the total number of reads in the sample to account for sequencing depth generated. We then normalized this ratio by the same ratio computed for the control sample (merge of all other protein samples) for each nucleotide.

Heatmaps were generated using Morpheus (https://software.broadinstitute.org/morpheus/). All values were included if they contained a significant 100nt window with a *p-value* < 0.001 (see above) and minimum enrichment of 3-fold above the control sample.

##### Gene ontology analysis

The 66 non-N enriched mRNAs were analyzed against the Gene Ontology Biological Processes and Reactome gene sets using the Molecular Signatures Database (MSigDB) (Liberzon et al., 2015). Significantly enriched gene sets with an FDR < 0.05 were used. To ensure that significant gene sets were not being driven by the multiple ribosomal proteins or histone proteins, these analyses were also carried out excluding these proteins.

#### Splicing analysis of 5EU data

Sequenced reads were demultiplexed according to barcoded RNA adaptor sequences ligated to each respective sample. Trimmomatic (https://github.com/timflutre/trimmomatic) was used to remove any contaminating Illumina primer sequences in the reads and low quality reads. Demultiplexed and trimmed files were then aligned to a hg19 reference genome using the splice-aware STAR aligner (https://github.com/alexdobin/STAR). Alignments were then deduplicated for PCR duplicates using PICARD MarkDuplicates (https://broadinstitute.github.io/picard/).

Aligned read-fragments were defined as read1 and read2 contained within a paired-end read fragment along with the insert between these two reads. We defined a set of high-quality represented isoforms per gene using the APPRIS database (Rodriguez et al., 2013). All read-fragments that spanned any 3′ splice site within an isoform of one of these genes was retained. For each 3′ splice site spanning fragment, we classified the read-fragment as a spliced fragment if it spanned an exon-exon junction (e.g., aligned entirely within 2 distinct exons) or an unspliced fragment if it spanned an intron-exon junction (e.g., one of the reads was contained -or partially contained – within the intron). For each isoform, we computed an unspliced ratio by counting the total number of reads that were classified as unspliced divided by the total number of read-fragments spanning 3′ splice sites within that gene. To ensure that the splicing ratio that we measured is a reliable metric and not inflated/deflated due to low read counts, we only included genes that contained at least 10 read-fragments in each sample and where the total number of reads in the control and sample conditions (when merged together) contained a significant number of reads to reliably measure a difference between the two groups as measured by a hypergeometric test (p < 0.01).

Because different genes contain different baseline splicing ratios due to gene length and coverage, we computed a change in the splicing ratio for each gene independently. To do this, we subtracted the unspliced ratio for each sample from the average unspliced ratio for that gene in all of the control samples. We plotted the overall distribution of these differences in splicing ratios as violin plots for each sample. If there is no change in splicing ratio, we would expect that some genes would have higher splicing ratios and others lower splicing ratios but that the overall distribution would be centered around 0.

#### Analysis of total RNA in SARS-CoV-2 infected samples

Total RNA-Seq libraries were generated from the same mock infected and SARS-CoV-2 virally infected Calu3 samples treated with 5EU. Prior to 5EU purification, total RNA was taken and an RNA-Seq library constructed as described above using barcoded RNA adapters. Cytoplasmic ribosomal RNAs (18S and 28S) were depleted using NEBNext ribosomal RNA depletion kit (NEB E6310L) per manufacturers recommendations. Demultiplexed reads were aligned using Bowtie2 (http://bowtie-bio.sourceforge.net/bowtie2/index.shtml) to custom genomes encoding classical noncoding RNAs (ncRNAs) or human messenger RNAs (mRNAs). Expression levels were computed for each mRNA by counting the total number of sequencing reads aligned to the mature mRNA. To normalize across the different libraries, we computed the read counts for each sample that align to non-spliced structural non-coding RNAs – excluding rRNA but including snRNAs, 7SL, 7SK, etc. We then divided each mRNA count by the sum of all ncRNA counts. This normalized value for each gene per sample was then converted into a fold-change by dividing this normalized value to the mean value for both mock infected samples. The fold change of each gene relative to mock was plotted across all mRNAs as a violin plot.

## Supplemental Information

Table S1. RNA Binding Map of SARS-CoV-2 Proteins, Related to Figure 1 A table of the enrichment level of each SARS-CoV-2 protein (rows) by all significantly enriched 100 nucleotide windows within human RNAs (columns). All bins with an enrichment *p-value* < 0.001 and enrichment > 3-fold for any of the viral proteins is reported.
