# Supplementary material for: SARS-CoV-2 Disrupts Splicing, Translation, and Protein Trafficking to Suppress Host Defenses
Source: Cell. 2020 Nov 25;183(5):1325–1339.e21. doi: 10.1016/j.cell.2020.10.004 (PMC7543886; doi:10.1016/j.cell.2020.10.004)
Supplement: Document S1. Tables S2–S5 [file mmc2.pdf]

Table S2: qPCR primers, Related to STAR Methods sections on Protein capture, siRNA experiments for SRP19 and SRP54, and Interferon stimulation experiments .

target	F primer	R primer
GAPDH	ATTCCACCCATGGCAAATTC	TGGGATTTCCATTGATGACAAG
IFN-beta	TTCAGTGTCAGAAGCTCCTGTGG	CTGCTTAATCTCCTCAGGGATGTCA
ISG54	CTTCCCAGTCTATCATCAACCTT	CCGTCGCTTCTAGCTATGTATCT
18S_1	CAT TCG AAC GTC TGC CCT AT	ACCCTGATTCCCCGTCAC
18S_2	ATT GGA GGG CAA GTC TGG T	CCAAGATCCAACTACGAGCTTT
LBR	AGT ATA GCC TTC GTC CAA GAA GA	CAAAGGTTCTCACTGCCAGTT
CALM3	GAC CAT TGA CTT CCC GGA GTT	GATGTAGCCATTCCCATCCTTG
SRP19	GCCGACCAGGACAGGTTTATT	TCTCTGTAGCTGTAGGATTTTCAAC
SRP54	TGGATTGCAAGGGAGTGGTAA	GGTCAAAAGCCCCTGCTCT

Table S3: Addgene ORFs, Related to STAR Methods sections on Generation of RNA binding maps, Cloning of expression constructs.

Gene Symbol	Synthesized CDS Name	Addgene clone number	URL	RRID
ORF1AB (split into 16 protein)	NSP1	141255	http://n2t.net/addgene:141255	RRID:Addgene_141255
	NSP2	141256	http://n2t.net/addgene:141256	RRID:Addgene_141256
	PLPRO (NSP3)	141257	http://n2t.net/addgene:141257	RRID:Addgene_141257
	NSP4	141258	http://n2t.net/addgene:141258	RRID:Addgene_141258
	NSP5	141259	http://n2t.net/addgene:141259	RRID:Addgene_141259
	NSP6	141260	http://n2t.net/addgene:141260	RRID:Addgene_141260
	NSP7	141261	http://n2t.net/addgene:141261	RRID:Addgene_141261
	NSP8	141262	http://n2t.net/addgene:141262	RRID:Addgene_141262
	NSP9	141263	http://n2t.net/addgene:141263	RRID:Addgene_141263
	NSP10	141264	http://n2t.net/addgene:141264	RRID:Addgene_141264
	NSP11	151991	http://n2t.net/addgene:151991	RRID:Addgene_151991
	RNA-pol (NSP12)	141265	http://n2t.net/addgene:141265	RRID:Addgene_141265
	Heli (NSP13)	141266	http://n2t.net/addgene:141266	RRID:Addgene_141266
	NSP14	141267	http://n2t.net/addgene:141267	RRID:Addgene_141267
	NSP15	141268	http://n2t.net/addgene:141268	RRID:Addgene_141268
	NSP16	141269	http://n2t.net/addgene:141269	RRID:Addgene_141269
ORF3A	3A	141271	http://n2t.net/addgene:141271	RRID:Addgene_141271
ORF3B	3B	141272	http://n2t.net/addgene:141272	RRID:Addgene_141272

E	E	141273	http://n2t.net/addgene:141273	RRID:Addgene_141273
M	M	141274	http://n2t.net/addgene:141274	RRID:Addgene_141274
ORF6	6	141275	http://n2t.net/addgene:141275	RRID:Addgene_141275
ORF7A	7A	141276	http://n2t.net/addgene:141276	RRID:Addgene_141276
ORF7B	7B	141277	http://n2t.net/addgene:141277	RRID:Addgene_141277
ORF8	8	141278	http://n2t.net/addgene:141278	RRID:Addgene_141278
ORF9B	9B	141280	http://n2t.net/addgene:141280	RRID:Addgene_141280
N	N	149330	http://n2t.net/addgene:149330	RRID:Addgene_149330

Table S4: Primers used in mRNA generation, Related to STAR Methods sections on *In vivo* translation assays and Leader NGFR measurements.

mRNA	F primer	R primer	template
- Leader mCherry	TAATACGACTCACTATAGGCTAGCACC ATGGTGA	CTACTACTACTTGTA CAGCTCGT	Leader mCherry Gblock
- Leader GFP	TAATACGACTCACTATAGGCTAGCACC ATGGCCCACATCGTGATGGT	CTTGTACAGCTCGTC CATGCCGA	Spytag-eGFP cDNA
+ Leader mCherry	TAATACGACTCACTATAGATTAAAGGT TTATACCTTCCCAGG	CTACTACTACTTGTA CAGCTCGT	Leader mCherry Gblock
+ Leader GFP	TAATACGACTCACTATAGATTAAAGGT TTATACCTTCCCAGGTAACAAACCAAC CAACTTTCGATCTCTTGTAGATCGCACC ATGGCCCACATCGTGATG	CTTGTACAGCTCGTC CATGCCGA	Spytag-eGFP cDNA
+5nt-SL1 mCherry	TAATACGACTCACTATAGAATTATTAA AGGTTTATACCTTCCCAGGTAACAAAC CAACCAACTTTCGATCTCTTGTAGATCG GCTAGCACCATGGTGA	CTACTACTACTTGTA CAGCTCGT	Leader mCherry Gblock
+SL1 mCherry	TAATACGACTCACTATAGATTAAAGGT TTATACCTTCCCAGGTAACAAACCGGC TAGCACCATGGTGA	CTACTACTACTTGTA CAGCTCGT	Leader mCherry Gblock
+SL2-SL1 mCherry	TAATACGACTCACTATAGATTAAAGAT CTCTTGTAGATCAACCAACTTTCGGTTT ATACCTTCCCAGGTAACAAACCGGCTA GCACCATGGTGA	CTACTACTACTTGTA CAGCTCGT	Leader mCherry Gblock
Nsp1	AAAAGGATCCATGGAGAGCCTGGTGCC	AAAACTCGAGTTAGC CACCGTTCAGTTCAC	Nsp1 mammalian expression vector
+ Leader NGFR	ATTAAAGGTTTATACCTTCCCAGGTAA CAAACCGGCTAGCACCATGGCCACAAC CATGGA	TTATCGATAAGCTTG GCTGCAG	pB-Halo-eGFP-IRES-NGFR mammalian expression vector

Table S5: Templates used in mRNA generation, Related to STAR Methods sections on *In vivo* translation assays.

Leader mCherry Gblock	TCAGTCGCGATCGAACCCTGGAATTCATTAAAGGTTTATACCTTCCCAGGT<br>AACAAACCAACCAACTTTCGATCTCTTGTAGATCTGTTCTCTAAACGAACG<br>GCTAGCACCATGGTGAGCAAGGGCGAGGAGGATAACATGGCCATCATCAA<br>GGAGTTCATGCGCTTCAAGGTGCACATGGAGGGCTCCGTGAACGGCCACG<br>AGTTCGAGATCGAGGGCGAGGGCGAGGGCCGCCCCTACGAGGGCACCCAG<br>ACCGCCAAGCTGAAGGTGACCAAGGGTGGCCCCCTGCCCTTCGCCTGGGA<br>CATCCTGTCCCCTCAGTTCATGTACGGCTCCAAGGCCTACGTGAAGCACCC<br>CGCCGACATCCCCGACTACTTGAAGCTGTCCTTCCCCGAGGGCTTCAAGTG<br>GGAGCGCGTGATGAACTTCGAGGACGGCGGCGTGGTGACCGTGACCCAGG<br>ACTCCTCCCTGCAGGACGGCGAGTTCATCTACAAGGTGAAGCTGCGCGGC<br>ACCAACTTCCCCTCCGACGGCCCCGTAATGCAGAAGAAGACCATGGGCTG<br>GGAGGCCTCCTCCGAGCGGATGTACCCCGAGGACGGCGCCCTGAAGGGCG<br>AGATCAAGCAGAGGCTGAAGCTGAAGGACGGCGGCCACTACGACGCTGA<br>GGTCAAGACCACCTACAAGGCCAAGAAGCCCGTGCAGCTGCCCGGCGCCT<br>ACAACGTCAACATCAAGTTGGACATCACCTCCCACAACGAGGACTACACC<br>ATCGTGGAACAGTACGAACGCGCCGAGGGCCGCCACTCCACCGGCGGCAT<br>GGACGAGCTGTACAAGTAGTAGTAGGCGGCCGCATAAAAATTAAAGA
Spytag-eGFP cDNA	AGCGGATAACAATTTCACACAGGAAACAGCTATGACCATGATTACGCCAA<br>GCTTATACGAAATTAATACGACTCACTATAGGGAGACCACAACGGTTTCCC<br>GAATTGTGAGCGGATAACAATAGAAATAATTTTGTTTAACTTTAAGAAGG<br>AGATATATCCATGGCCCACATCGTGATGGTGGACGCCTACAAGCCTACTAA<br>GGGCAGCGGCTCTAGAATGGCCACAACCATGGTGAGCAAGGGCGAGGAG<br>CTGTTCACCGGGGTGGTGCCCATCCTGGTCGAGCTGGACGGCGACGTAAA<br>CGGCCACAAGTTCAGCGTGTCTGGCGAGGGCGAGGGCGATGCCACCTACG<br>GCAAGCTGACCCTGAAGTTCATCTGCACCACCGGCAAGCTGCCCGTGCCCT<br>GGCCCACCCTCGTGACCACCCTGACCTACGGCGTGCAGTGCTTCAGCCGCT<br>ACCCCGACCACATGAAGCAGCACGACTTCTTCAAGTCCGCCATGCCCGAA<br>GGCTACGTCCAGGAGCGCACCATCTTCTTCAAGGACGACGGCAACTACAA<br>GACCCGCGCCGAGGTGAAGTTCGAGGGCGACACCCTGGTGAACCGCATCG<br>AGCTGAAGGGCATCGACTTCAAGGAGGACGGCAACATCCTGGGGCACAAG<br>CTGGAGTACAACTACAACAGCCACAACGTCTATATCATGGCCGACAAGCA<br>GAAGAACGGCATCAAGGCGAACTTCAAGATCCGCCACAACATCGAGGACG<br>GCAGCGTGCAGCTCGCCGACCACTACCAGCAGAACACCCCCATCGGCGAC<br>GGCCCCGTGCTGCTGCCCGACAACCACTACCTGAGCACCCAGTCCGCCCTG<br>AGCAAAGACCCCAACGAGAAGCGCGATCACATGGTCCTGCTGGAGTTCGT<br>GACCGCCGCCGGGATCACTCTCGGCATGGACGAGCTGTACAAGGGATTCG<br>GATCCGAACAAAAACTCATCTCAGAAGAGGATCTGGCGGCCGCTGGATCT<br>GGTGGCCAGAAGCAAGCTGAAGAGGCGGCAGCGAAAGCGGCGGCAGATG<br>CTAAAGCGAAGGCCGAAGCAGATGCTAAAGCTGCGGAAGAAGCAGCGAA<br>GAAAGCGGCTGCAGACGCAAAGAAAAAAGCAGAAGCAGAAGCCGCCAAA<br>GCCGCAGCCGAAGCGCAGAAAAAAGCCGAGGCAGCCGCTGCGGCACTGA<br>AGAAGAAAGCGGAAGCGGCAGAAGCAGCTGCAGCTGAAGCAAGAAAGAA<br>AGCGGCAACTGAAACCGCACACCTTACTGGTGTGCGGGGCATCCCGACAT<br>GGACGTTGAATTATGAATTCACTGGCCGTCGTTTTACA
